# Supplementary material for: Inducible Degradation of Target Proteins through a Tractable Affinity-Directed Protein Missile System
Source: Cell Chem Biol. 2020 Sep 17;27(9):1164–1180.e5. doi: 10.1016/j.chembiol.2020.06.013 (PMC7505680; doi:10.1016/j.chembiol.2020.06.013)
Supplement: Document S1. Figures S1–S4 [file mmc1.pdf]

Cell Chemical Biology, Volume *27*

Supplemental Information

Inducible Degradation of Target Proteins through a Tractable Affinity-Directed Protein Missile System

Luke M. Simpson, Thomas J. Macartney, Alice Nardin, Luke J. Fulcher, Sascha Röth, Andrea Testa, Chiara Maniaci, Alessio Ciulli, Ian G. Ganley, and Gopal P. Sapkota

Supplemental Information

Inducible degradation of target proteins through a tractable Affinity-directed PROtein Missile (AdPROM) system

Luke M. Simpson, Thomas J. Macartney, Alice Nardin, Luke J. Fulcher, Sascha Röth, Andrea Testa, Chiara Maniaci, Alessio Ciulli, Ian G. Ganley and Gopal P. Sapkota

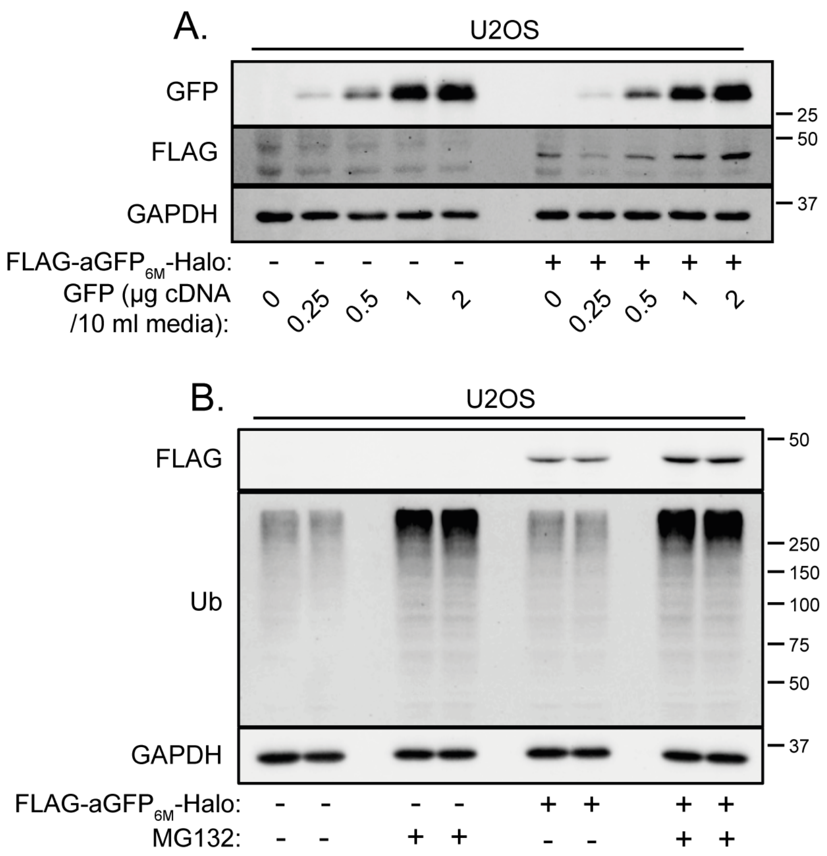

Figure S1, related to Figure 1. FLAG-aGFP$_{6M}$-Halo levels are controlled by GFP protein abundance. (**A**) GFP was transiently expressed with increasing cDNA concentrations (0-2 µg per 10 ml media) in U2OS FLAG-empty and FLAG-aGFP$_{6M}$-Halo expressing cells. (**B**) U2OS FLAG-empty and FLAG-aGFP$_{6M}$-Halo expressing cells were treated with 20 µM MG132 proteasome inhibitor for 6 hr as indicated. For both (**A**) and (**B**), extracts were resolved by SDS-PAGE and transferred on to PVDF membranes, which were subjected to immunoblotting with indicated antibodies.

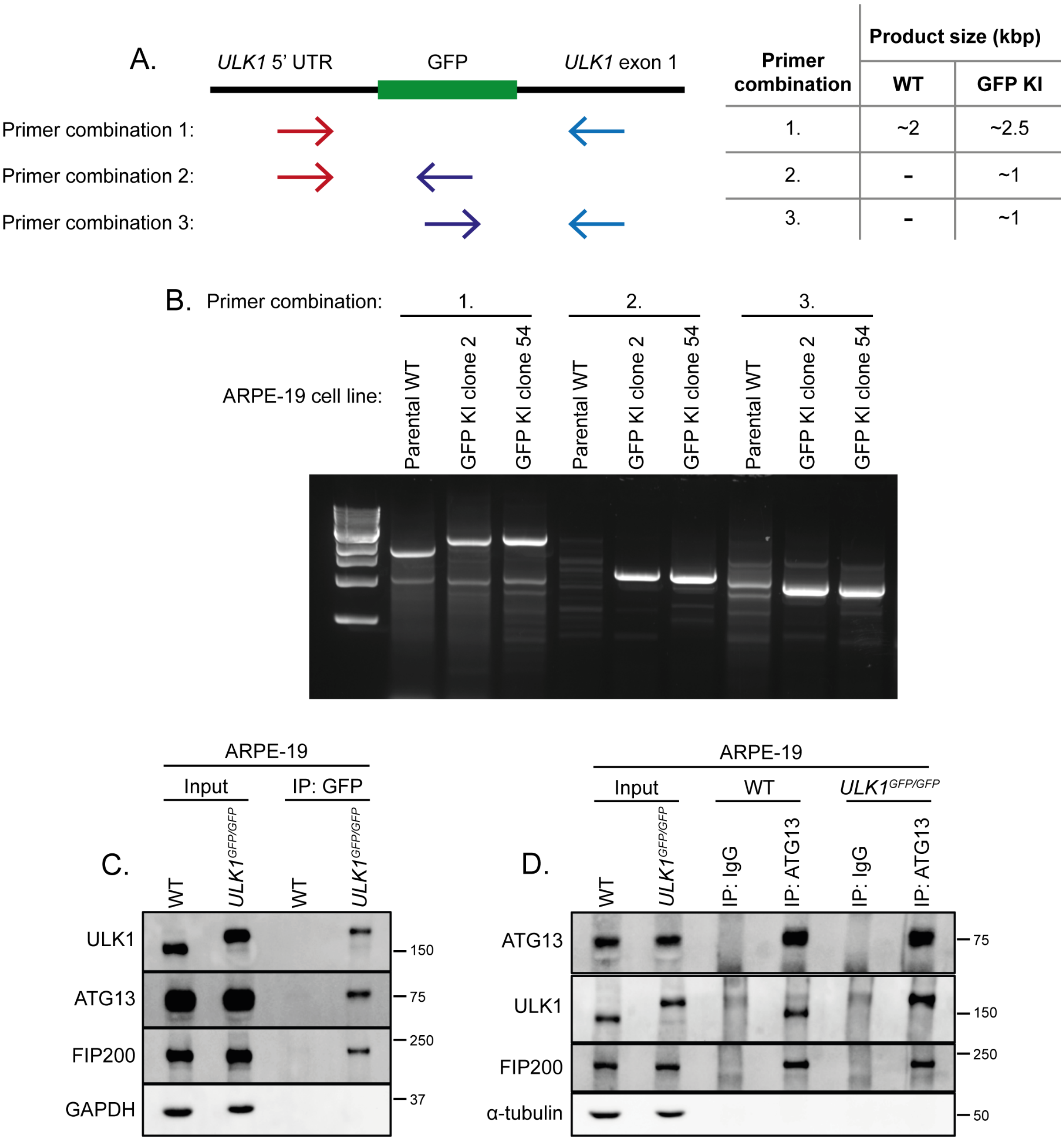

Figure S2, related to Figure 1. Characterisation of ARPE-19 ULK1 GFP knockin (KI) (*ULK1$^{GFP/GFP}$*) cells. (**A**) Primers were designed for analysis of the endogenous *ULK1* locus (primer combination 1) and internal primers to recognise the incorporated GFP-tag (primer combination 2 and 3). (**B**) Agarose gel analysis of ARPE-19 parental WT and ULK1 GFP KI cell line clones 2 and 54. In both clones, an electrophoretic mobility shift is observed in the PCR products of primer combination 1. With both primer combination 2 and 3, no PCR product was observed in parental WT cells, but can be detected for both clone 2 and 54. Clone 54 was used for subsequent experiments. (**C**) ARPE-19 WT and *ULK1$^{GFP/GFP}$* cells were lysed and subjected to GFP TRAP immunoprecipitation (IP). ULK1 complex components including GFP-ULK1, ATG13 and FIP200 co-precipitated with GFP-ULK1 from *ULK1$^{GFP/GFP}$* cell extracts. (**D**) ARPE-19 WT and *ULK1$^{GFP/GFP}$* cells were lysed and subjected to ATG13 or IgG IP as indicated. ULK1 complex components including ATG13, ULK1 and FIP200 co-precipitated with ATG13 from WT and *ULK1$^{GFP/GFP}$* cell extracts. For both (**C**) and (**D**), extracts and IPs were resolved by SDS-PAGE and transferred on to PVDF membranes, which were subjected to immunoblotting with indicated antibodies.

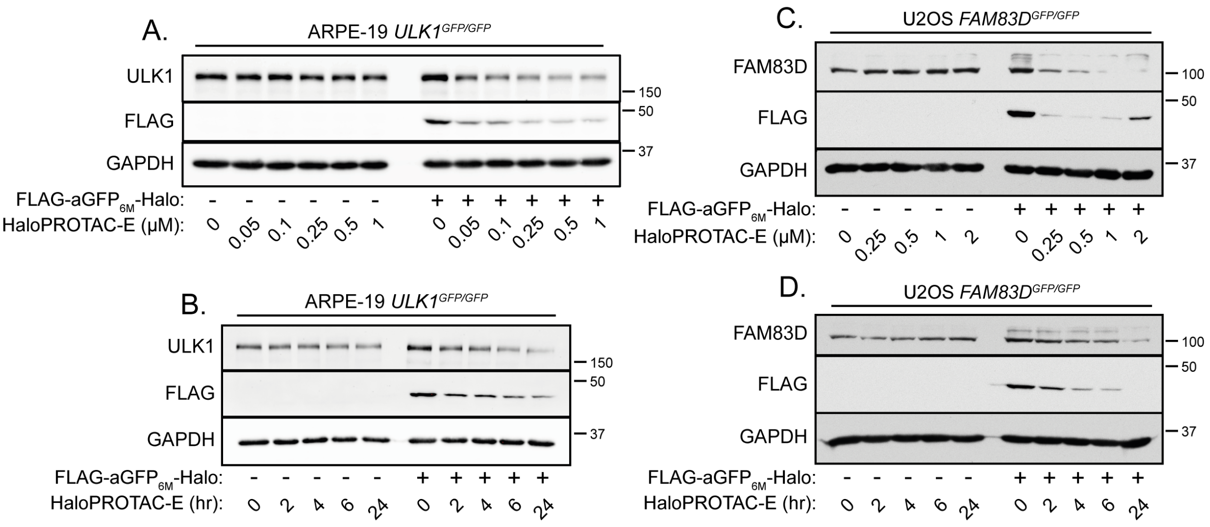

Figure S3, related to Figure 1. GFP-ULK1 and FAM83D-GFP are degraded with HaloPROTAC-E in cells expressing FLAG-aGFP$_{6M}$-Halo. (**A**) ARPE-19 *ULK1$^{GFP/GFP}$* FLAG-empty and FLAG-aGFP$_{6M}$-Halo expressing cells were treated with increasing concentrations of HaloPROTAC-E (0-1 μM) for 24 hr as indicated. (**B**) ARPE-19 *ULK1$^{GFP/GFP}$* FLAG-empty and FLAG-aGFP$_{6M}$-Halo expressing cells were treated with 250 nM HaloPROTAC-E for indicated times (0-24 hr). (**C**) U2OS *FAM83D$^{GFP/GFP}$* FLAG-empty and FLAG-aGFP$_{6M}$-Halo expressing cells were treated with increasing concentrations of HaloPROTAC-E (0-2 μM) for 24 hr. (**D**) U2OS *FAM83D$^{GFP/GFP}$* FLAG-empty and FLAG-aGFP$_{6M}$-Halo expressing cells were treated with 1 μM HaloPROTAC-E for indicated times (0-24 hr). For all experiments, extracts were resolved by SDS-PAGE and transferred on to PVDF membranes, which were subjected to immunoblotting with indicated antibodies.

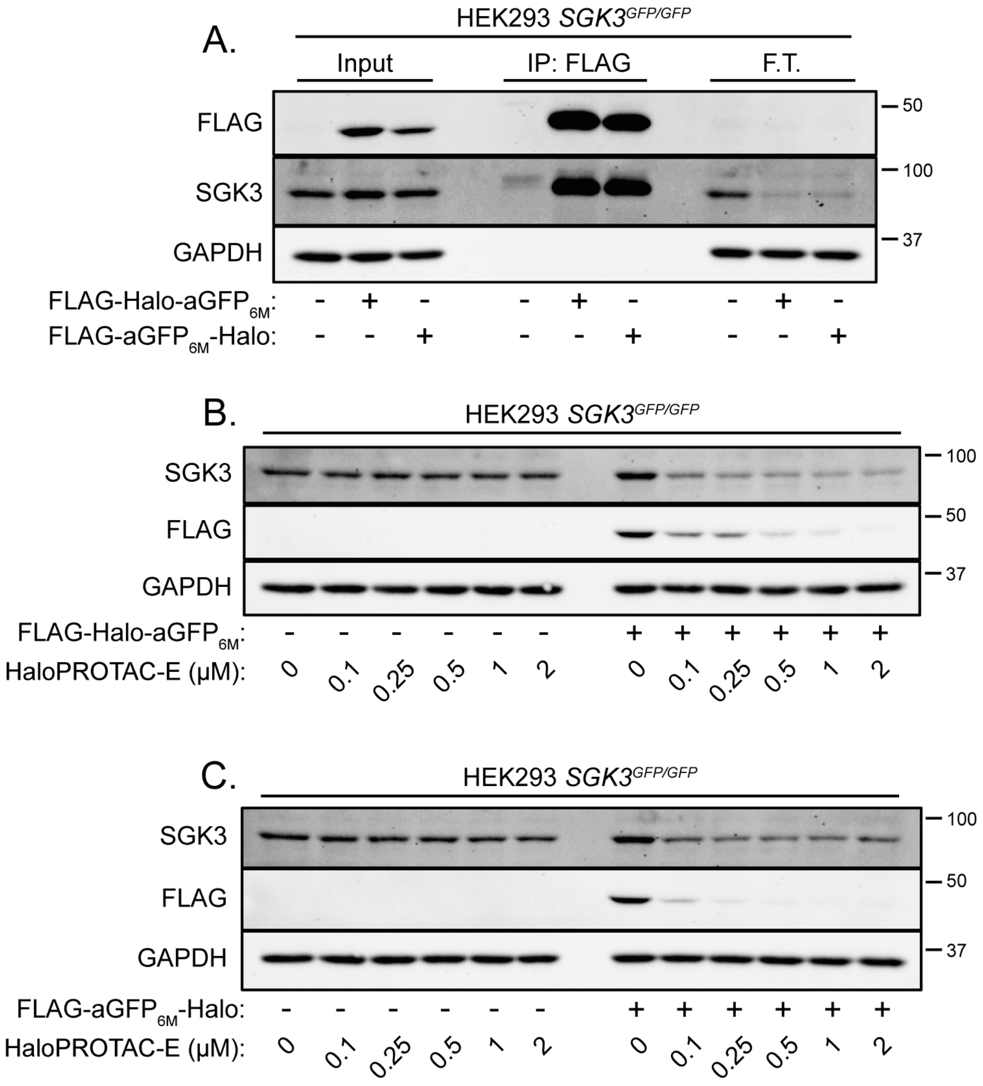

Figure S4, related to Figure 3. SGK3-GFP is degraded with HaloPROTAC-E in FLAG-Halo-aGFP$_{6M}$ and FLAG-aGFP$_{6M}$-Halo expressing cells. (**A**) HEK293 *SGK3*$^{GFP/GFP}$ FLAG-empty, FLAG-Halo-aGFP$_{6M}$ and FLAG-aGFP$_{6M}$-Halo expressing cells were lysed and subjected to immunoprecipitation (IP) with anti-FLAG M2 resin. F.T. = post-IP flow-through extract. HEK293 *SGK3*$^{GFP/GFP}$ FLAG-empty, FLAG-Halo-aGFP$_{6M}$ (**B**) and FLAG-aGFP$_{6M}$-Halo (**C**) expressing cells were treated with increasing concentrations of HaloPROTAC-E (0-2 μM) for 24 hr as indicated. In all cases, extracts and IPs were resolved by SDS-PAGE and transferred on to PVDF membranes, which were subjected to immunoblotting with indicated antibodies.
