# Supplementary material for: Inducible Degradation of Target Proteins through a Tractable Affinity-Directed Protein Missile System
Source: Cell Chem Biol. 2020 Sep 17;27(9):1164–1180.e5. doi: 10.1016/j.chembiol.2020.06.013 (PMC7505680; doi:10.1016/j.chembiol.2020.06.013)
Supplement: Document S2. Article plus Supplemental Information [file mmc2.pdf]

Article

Cell Chemical Biology

Inducible Degradation of Target Proteins through a Tractable Affinity-Directed Protein Missile System

Graphical Abstract

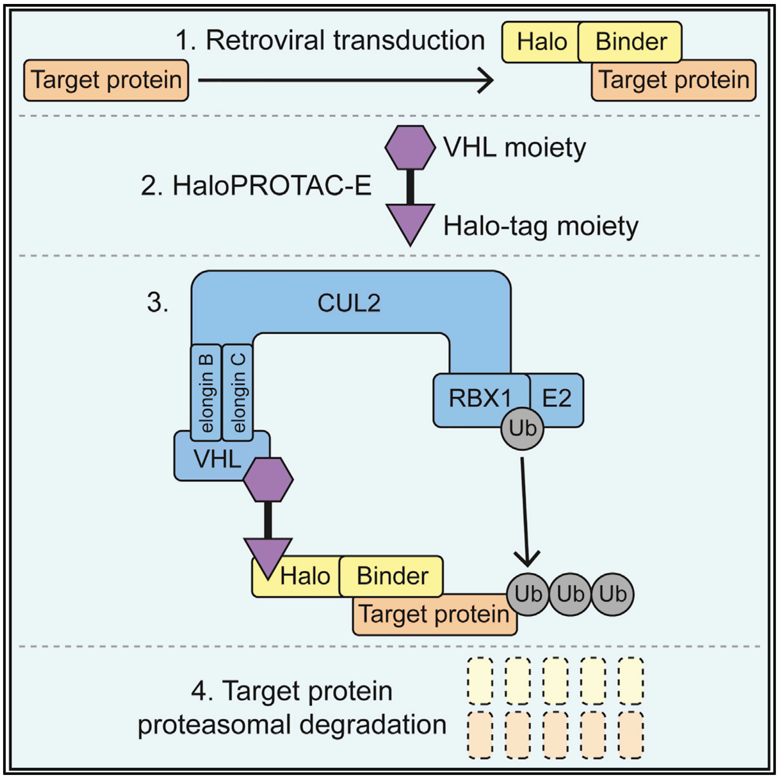

Highlights

- Ligand (L)-inducible AdPROM consists of Halo conjugated to a target protein binder
- Target protein is degraded with HaloPROTAC in cells expressing L-AdPROM construct
- HaloPROTAC-mediated target protein degradation using L-AdPROM system is reversible
- Degradation using HaloPROTAC L-AdPROM impacts target protein biological function

Authors

Luke M. Simpson,
Thomas J. Macartney, Alice Nardin, ...,
Alessio Ciulli, Ian G. Ganley,
Gopal P. Sapkota

Correspondence

g.sapkota@dundee.ac.uk

In Brief

Simpson et al. combine Halo-tag/VHL-recruiting proteolysis-targeting chimera (HaloPROTAC) technology with high-affinity small polypeptide binders to develop a ligand-inducible degradation system (L-AdPROM) for target proteins of interest (POI). In cells expressing a Halo-POI binder, target protein degradation occurs only in the presence of the HaloPROTAC.

Simpson et al., 2020, Cell Chemical Biology *27*, 1164–1180
September 17, 2020 © 2020 The Authors. Published by Elsevier Ltd.
https://doi.org/10.1016/j.chembiol.2020.06.013

CellPress

Cell Chemical Biology

Article

Inducible Degradation of Target Proteins through a Tractable Affinity-Directed Protein Missile System

Luke M. Simpson,[1] Thomas J. Macartney,[1] Alice Nardin,[1] Luke J. Fulcher,[1,3] Sascha Röth,[1] Andrea Testa,[2,4] Chiara Maniaci,[2,5] Alessio Ciulli,[2] Ian G. Ganley,[1] and Gopal P. Sapkota[1,6,*]
[1]Medical Research Council (MRC) Protein Phosphorylation & Ubiquitylation Unit, School of Life Sciences, University of Dundee, Dundee DD1 5EH, UK
[2]Division of Biological Chemistry & Drug Discovery, School of Life Sciences, University of Dundee, Dundee DD1 5EH, UK
[3]Department of Biochemistry, University of Oxford, South Parks Road, Oxford OX1 3QU, UK
[4]Amphista Therapeutics Ltd, Bo'Ness Road, Newhouse ML1 5UH, UK
[5]School of Natural & Environmental Sciences, Chemistry Bedson Building, Kings Road, Newcastle University, Newcastle Upon Tyne NE1 7RU, UK
[6]Lead Contact
*Correspondence: g.sapkota@dundee.ac.uk
https://doi.org/10.1016/j.chembiol.2020.06.013

SUMMARY

The affinity-directed protein missile (AdPROM) system utilizes specific polypeptide binders of intracellular proteins of interest (POIs) conjugated to an E3 ubiquitin ligase moiety to enable targeted proteolysis of the POI. However, a chemically tuneable AdPROM system is more desirable. Here, we use Halo-tag/VHL-recruiting proteolysis-targeting chimera (HaloPROTAC) technology to develop a ligand-inducible AdPROM (L-AdPROM) system. When we express an L-AdPROM construct consisting of an anti-GFP nanobody conjugated to the Halo-tag, we achieve robust degradation of GFP-tagged POIs only upon treatment of cells with the HaloPROTAC. For GFP-tagged POIs, ULK1, FAM83D, and SGK3 were knocked in with a GFP-tag using CRISPR/Cas9. By substituting the anti-GFP nanobody for a monobody that binds H- and K-RAS, we achieve robust degradation of unmodified endogenous RAS proteins only in the presence of the HaloPROTAC. Through substitution of the polypeptide binder, the highly versatile L-AdPROM system is useful for the inducible degradation of potentially any intracellular POI.

INTRODUCTION

Developments in RNA interference (RNAi) and CRISPR/Cas9 technologies have enabled the manipulation of specific proteins of interest (POIs) to study and understand their biological functions (Elbashir et al., 2001; Cong et al., 2013; Doudna and Charpentier, 2014; Sander and Joung, 2014). However, as RNAi indirectly depletes target protein expression through the disruption of messenger RNA, and therefore is reliant on natural protein turnover, it can be inefficient and time-consuming, especially when targeting proteins with slow turnover rates (Elbashir et al., 2001; Jansen et al., 2007; Smoak et al., 2016). In addition, RNAi has been shown to introduce off-target effects (Rossi et al., 2015). The generation of target protein knockout (KO) cell lines using CRISPR/Cas9 genome editing technology can be time consuming and is not feasible for every target protein, particularly when targeting genes that are essential for cell survival or proliferation (Wang et al., 2015), or for every cell line. Therefore, advances in targeted protein degradation technologies could overcome these current limitations.

The ubiquitin-proteasome system (UPS) plays a fundamental role in the degradation of proteins to maintain cellular homeostasis (Roos-Mattjus and Sistonen, 2004; Pines and Lindon, 2005). Through sequential actions of the E1 ubiquitin-activating enzyme, E2 ubiquitin-conjugating enzymes, and E3 ubiquitin ligases, target proteins are covalently labeled with ubiquitin chains, marking them for recognition and degradation by the proteasome (Scheffner et al., 1995). The Cullin (CUL) really interesting new gene (RING) E3 ligase (CRL) family plays a fundamental role in regulating protein turnover in cells through the UPS (Wenzel et al., 2011; Zhao et al., 2012). CRLs are activated through NEDDylation, where the small ubiquitin-like modifier NEDD8 (neural precursor cell expressed developmentally down-regulated protein 8) is covalently attached to a lysine residue of the CUL (Soucy et al., 2009). CUL2-CRL is in a complex with Elongin B and C adaptors, the substrate receptor von Hippel-Lindau (VHL) protein and the RING-box protein 1 (RBX1) E3 ligase (Cardote et al., 2017). Under normoxic conditions, VHL binds to hydroxy-proline-modified hypoxia-inducible factor 1α (HIF1α) and brings HIF1α in close proximity to RBX1 for its

 © 2020 The Authors. Published by Elsevier Ltd.
This is an open access article under the CC BY license (http://creativecommons.org/licenses/by/4.0/).

CellPress
OPEN ACCESS

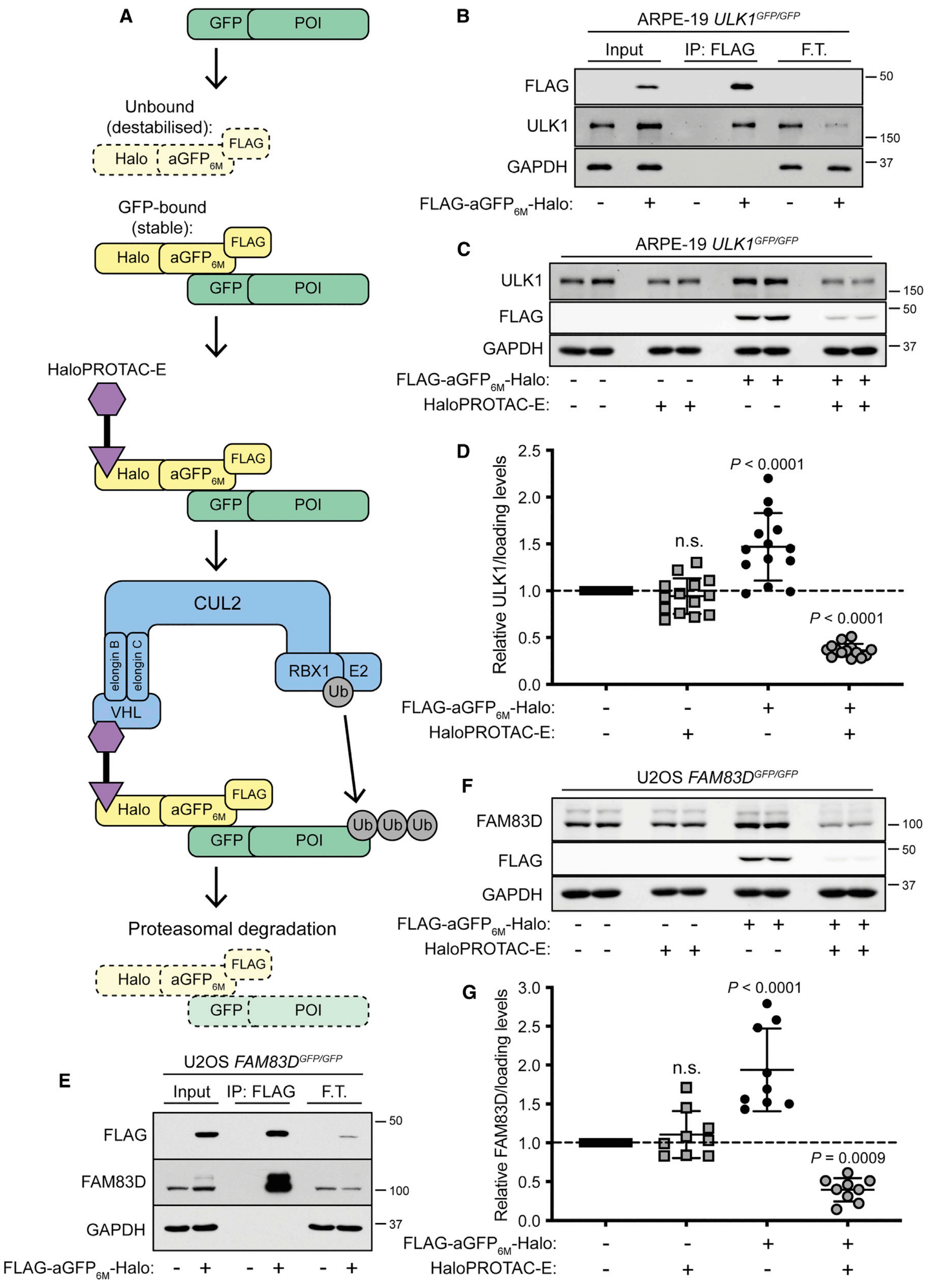

(legend on next page)

ubiquitylation and subsequent degradation by the proteasome (Ohh et al., 2000; Ivan et al., 2001; Jaakkola et al., 2001).

Through the exploitation of the endogenous CUL2-CRL machinery and small polypeptide binders of target proteins, we recently reported an efficient affinity-directed protein missile (AdPROM) system for the proteolysis of endogenous target proteins (Fulcher et al., 2016, 2017). AdPROM was engineered with VHL tethered to, for example, an anti-GFP nanobody (aGFP) for either constitutive or tetracycline (Tet)-inducible degradation of GFP-tagged proteins knocked in using CRISPR/Cas9. However, Tet-inducible AdPROM necessitates the generation of multicomponent cell lines, is often leaky, and relies on transcription and translation of the AdPROM constructs, thereby limiting rapid target protein degradation.

To overcome these limitations, a robust tractable AdPROM system able to achieve rapid and chemically tuneable degradation of target proteins is desirable. Small-molecule approaches, including the use of proteolysis-targeting chimeras (PROTACs), for rapid target protein degradation have been previously reported (Bondeson et al., 2015; Bondeson and Crews, 2017). PROTACs are heterobifunctional molecules that bring a target protein into spatial proximity with an E3 ubiquitin ligase to trigger target ubiquitylation and subsequent proteasomal degradation (Sakamoto et al., 2001; Lucas and Ciulli, 2017; Toure and Crews, 2016). PROTACs that hijack CUL2-CRL using derivatives of the VHL ligand's hydroxyproline have been developed to induce degradation of the bromodomain (BRD) and extra-terminal domain proteins BRD2, BRD3, and BRD4, and the estrogen-related receptor α (ERRα) in cells and *in vivo* (Bondeson et al., 2015; Zengerle et al., 2015; Gadd et al., 2017). Halo-based PROTACs that simultaneously bind the Halo-tag (Los et al., 2008; Ohana et al., 2009) and VHL through distinct binding moieties have previously been described for the inducible degradation of overexpressed Halo-tagged target proteins (Buckley et al., 2015; Tomoshige et al., 2016). More recently, HaloPROTAC-E was developed for the inducible degradation of target proteins consisting of a Halo-tag knocked in using CRISPR/Cas9 technology (Tovell et al., 2019a). However, highlighting the difficulty of achieving homozygous integration of a non-fluorescent Halo-tag onto target genes, it was only possible to isolate a clone where Halo-tag was inserted on one allele of SGK3 (serum and glucocorticoid-induced protein kinase 3) (Tovell et al., 2019a), whereas multiple clones for the homozygous integration of a GFP-tag on SGK3 were achieved (Malik et al., 2018). By expressing an AdPROM construct consisting of a target protein-specific polypeptide binder conjugated to the Halo-tag, we sought to utilize HaloPROTAC-E for the inducible degradation of target proteins.

RESULTS

GFP-ULK1 and FAM83D-GFP Are Degraded with HaloPROTAC-E in Cells Expressing FLAG-aGFP$_{6M}$-Halo

First, we developed a ligand-inducible AdPROM (L-AdPROM) construct, consisting of aGFP conjugated to the Halo-tag and tagged with a FLAG reporter, for the degradation of GFP-tagged POIs only in the presence of HaloPROTAC-E (Figure 1A). Rather than use constructs that yield overexpression of aGFP relative to the target, an antigen-stabilized aGFP mutant (aGFP$_{6M}$) was utilized (Tang et al., 2016). In this case, aGFP$_{6M}$ is only stable when bound to GFP and destabilized and degraded when unbound, thereby maintaining homeostatic FLAG-aGFP$_{6M}$-Halo levels close to a 1:1 ratio to POI-GFP. In the presence of POI-GFP, FLAG-aGFP$_{6M}$-Halo binds POI-GFP with high affinity. Treating these cells with HaloPROTAC-E then recruits FLAG-aGFP$_{6M}$-Halo bound to POI-GFP to VHL. Consequently, the POI-GFP:FLAG-aGFP$_{6M}$-Halo complex is ubiquitylated by the CUL2-CRL machinery and degraded by the proteasome.

To analyze the expression of FLAG-aGFP$_{6M}$-Halo in the absence or presence of GFP, GFP was transiently expressed with increasing concentrations of cDNA in both U2OS wild-type (WT) cells and those transduced with retrovirus encoding FLAG-aGFP$_{6M}$-Halo (Figure S1A). As expected, GFP protein expression in both cell lines increased with increasing concentrations of cDNA used for transfection. In cells transduced with FLAG-aGFP$_{6M}$-Halo, low levels of FLAG-aGFP$_{6M}$-Halo protein expression were detected in untransfected control cells, which increased with increasing levels of GFP, suggesting that the antigen-dependent nature of aGFP$_{6M}$ ensures that the homeostatic level of FLAG-aGFP$_{6M}$-Halo is controlled by POI-GFP protein abundance. To determine whether unbound FLAG-aGFP$_{6M}$-Halo destabilization was facilitated by the proteasome, U2OS FLAG-aGFP$_{6M}$-Halo-expressing cells were treated with the proteasome inhibitor MG132 (Figure S1B). In MG132-treated cells, an increase in poly-ubiquitylated conjugates (Ub) was observed compared with DMSO-treated controls, suggesting successful inhibition of the proteasome. Under these conditions, stabilization of FLAG-aGFP$_{6M}$-Halo was observed in MG132-treated FLAG-aGFP$_{6M}$-Halo-expressing cells relative to DMSO-treated controls, suggesting that unbound FLAG-aGFP$_{6M}$-Halo destabilization is facilitated by the proteasome.

For initial analyses of the FLAG-aGFP$_{6M}$-Halo L-AdPROM system, FLAG-aGFP$_{6M}$-Halo was expressed by retroviral transduction in ARPE-19 ULK1 GFP knockin (KI) (*ULK1*$^{GFP/GFP}$) cells, which were generated using CRISPR/Cas9 technology (Figure S2). ULK1 (unc-51-like kinase 1) is a serine/threonine protein kinase that plays a key role in the initiation of autophagy, a crucial

Figure 1. GFP-ULK1 and FAM83D-GFP Are Degraded with HaloPROTAC-E in Cells Expressing FLAG-aGFP$_{6M}$-Halo

(A) Schematic representation of FLAG-aGFP$_{6M}$-Halo HaloPROTAC L-AdPROM system.

(B and E) ARPE-19 *ULK1*$^{GFP/GFP}$ (B) and U2OS *FAM83D*$^{GFP/GFP}$ (E) FLAG-empty and FLAG-aGFP$_{6M}$-Halo-expressing cells were lysed and subjected to immunoprecipitation (IP) with anti-FLAG M2 resin. F.T., post-IP flow-through extract.

(C) ARPE-19 *ULK1*$^{GFP/GFP}$ FLAG-empty and FLAG-aGFP$_{6M}$-Halo-expressing cells were treated with 250 nM HaloPROTAC-E for 24 h.

(D) Quantification of relative GFP-ULK1 protein levels from (C) normalized to loading control ± SD of n = 14 independent experiments.

(F) U2OS *FAM83D*$^{GFP/GFP}$ FLAG-empty and FLAG-aGFP$_{6M}$-Halo-expressing cells were treated with 1 μM HaloPROTAC-E for 24 h.

(G) Quantification of relative FAM83D-GFP protein levels from (F) normalized to loading control ±SD of n = 9 independent experiments.

Statistical analyses were carried out by one-way analysis of variance using Dunnett's post-test; n.s., not significant. For (B), (C), (E), and (F), extracts and IPs were resolved by SDS-PAGE and transferred on to PVDF membranes, which were subjected to immunoblotting with indicated antibodies.

lysosomal degradation pathway that serves as a quality control mechanism to recycle damaged, toxic, or excess cellular components and maintain protein synthesis under starvation conditions (Zachari and Ganley, 2017). Anti-FLAG immunoprecipitations (IPs) from *ULK1*$^{GFP/GFP}$ FLAG-aGFP$_{6M}$-Halo expressing, but not FLAG-empty control, cell extracts co-precipitated GFP-ULK1 (Figure 1B), which was depleted from flow-through extracts, confirming that FLAG-aGFP$_{6M}$-Halo interacts with GFP-ULK1. To assess HaloPROTAC-E-mediated GFP-ULK1 degradation in these cells, both *ULK1*$^{GFP/GFP}$ FLAG-empty control and FLAG-aGFP$_{6M}$-Halo-expressing cells were treated with increasing concentrations of HaloPROTAC-E (0.05–1 μM) for 24 h (Figure S3A). No change in GFP-ULK1 levels were observed in HaloPROTAC-E-treated FLAG-empty cells. However, in FLAG-aGFP$_{6M}$-Halo-expressing cells, a reduction in GFP-ULK1 levels was observed with 0.25, 0.5, and 1 μM HaloPROTAC-E. Parallel degradation of FLAG-aGFP$_{6M}$-Halo was also observed at these HaloPROTAC-E concentrations, either due to co-degradation or destabilizing mutations triggering proteolysis once the bound cargo was degraded. To analyze the time-dependent degradation of GFP-ULK1 with HaloPROTAC-E, *ULK1*$^{GFP/GFP}$ FLAG-empty control and FLAG-aGFP$_{6M}$-Halo-expressing cells were treated with 250 nM HaloPROTAC-E for 2, 4, 6, and 24 h (Figure S3B). Both GFP-ULK1 and FLAG displayed time-dependent degradation upon treatment with HaloPROTAC-E only in FLAG-aGFP$_{6M}$-Halo-expressing cells, with optimal degradation achieved after 24 h. Following HaloPROTAC-E treatment optimization, *ULK1*$^{GFP/GFP}$ FLAG-empty control and FLAG-aGFP$_{6M}$-Halo-expressing cells were treated with 250 nM HaloPROTAC-E for 24 h and GFP-ULK1 protein levels were quantified (Figures 1C and 1D). Although a slight but significant stabilization of GFP-ULK1 was observed upon FLAG-aGFP$_{6M}$-Halo expression compared with DMSO-treated FLAG-empty control cells, a mean 65% reduction in GFP-ULK1 protein levels was observed with HaloPROTAC-E.

To determine the applicability and versatility of the HaloPROTAC L-AdPROM system, FLAG-aGFP$_{6M}$-Halo was expressed by retroviral transduction in U2OS FAM83D GFP KI (*FAM83D*$^{GFP/GFP}$) cells (Fulcher et al., 2019), and HaloPROTAC-E-mediated FAM83D-GFP degradation assessed. FAM83D belongs to the FAMily with sequence similarity 83 (FAM83) family of poorly characterized proteins (Bozatzi and Sapkota, 2018; Fulcher et al., 2018). FAM83D is required for the recruitment of casein kinase 1α (CK1α) to the mitotic spindle to orchestrate proper spindle positioning and timely cell division (Fulcher et al., 2019). FAM83D-GFP co-precipitated only with anti-FLAG IPs from *FAM83D*$^{GFP/GFP}$ FLAG-aGFP$_{6M}$-Halo-expressing cells (Figure 1E), and not FLAG-empty control cells, confirming that FLAG-aGFP$_{6M}$-Halo interacts with FAM83D-GFP. Treatment of *FAM83D*$^{GFP/GFP}$ FLAG-aGFP$_{6M}$-Halo-expressing cells with increasing concentrations of HaloPROTAC-E (0.25–2 μM) for 24 h resulted in a decrease in FAM83D-GFP levels in a dose-dependent manner (Figure S3C), while no degradation was evident in FLAG-empty control cells. Optimal FAM83D-GFP degradation was observed with 1 μM HaloPROTAC-E, with stabilization observed at 2 μM. This high-dose hook effect is where degradation is decreased at high compound concentrations as the formation of binary complexes outcompetes the active ternary complexes (Douglass et al., 2013). To analyze FAM83D-GFP degradation kinetics with HaloPROTAC-E, *FAM83D*$^{GFP/GFP}$ FLAG-empty, and FLAG-aGFP$_{6M}$-Halo-expressing cells were treated with 1 μM HaloPROTAC-E for 2, 4, 6, and 24 h (Figure S3D). In HaloPROTAC-E-treated FLAG-aGFP$_{6M}$-Halo-expressing cells, FAM83D-GFP was degraded in a time-dependent manner, with optimal degradation achieved after 24 h. FAM83D-GFP levels were then quantified 24 h after 1 μM HaloPROTAC-E treatment (Figures 1F and 1G). Although a slight but significant stabilization of FAM83D-GFP was observed upon FLAG-aGFP$_{6M}$-Halo expression compared with DMSO-treated FLAG-empty control cells, a mean 65% reduction in FAM83D-GFP protein levels was observed with HaloPROTAC-E.

Characterization of HaloPROTAC-E L-AdPROM-Mediated GFP-ULK1 and FAM83D-GFP Degradation

To determine whether HaloPROTAC-E-mediated degradation of GFP-ULK1 and FAM83D-GFP in FLAG-aGFP$_{6M}$-Halo-expressing cells requires the binding of HaloPROTAC-E to Halo, an FLAG-aGFP$_{6M}$-Halo^{D106A} mutant that cannot bind the ligand (Neklesa et al., 2011) was expressed in ARPE-19 *ULK1*$^{GFP/GFP}$ (Figure 2A) and U2OS *FAM83D*$^{GFP/GFP}$ (Figure 2B) cells by retroviral transduction. In these cells, HaloPROTAC-E treatment failed to degrade either GFP-ULK1 (Figure 2A) or FAM83D-GFP (Figure 2B), suggesting that the HaloPROTAC-E:Halo interaction is necessary for GFP-ULK1 and FAM83D-GFP degradation in FLAG-aGFP$_{6M}$-Halo-expressing cells. Next, to assess whether HaloPROTAC-E-mediated degradation of GFP-ULK1 and FAM83D-GFP in FLAG-aGFP$_{6M}$-Halo-expressing cells requires the binding of HaloPROTAC-E to VHL, a competition assay with the VHL inhibitor VH298 (Frost et al., 2016), which the VHL warhead of HaloPROTAC-E is based on, was established (Figures 2C and 2D). VH298 not only stabilized HIF1α protein levels, thereby confirming the inhibition of VHL, but also inhibited the degradation of both GFP-ULK1 (Figure 2C) and FAM83D-GFP (Figure 2D) caused by HaloPROTAC-E in the respective FLAG-aGFP$_{6M}$-Halo-expressing cells. These data suggest that HaloPROTAC-E successfully binds VHL to mediate GFP-ULK1 and FAM83D-GFP degradation in FLAG-aGFP$_{6M}$-Halo-expressing cells. On the other hand, neither HaloPROTAC-E treatment nor FLAG-aGFP$_{6M}$-Halo expression in cells influenced HIF1α levels (Figures 2C and 2D), suggesting that they do not interfere with the endogenous VHL-CUL2-CRL machinery. To determine whether HaloPROTAC-E-mediated GFP-ULK1 and FAM83D-GFP degradation was facilitated by the CUL-CRL machinery, the pan-CUL NEDDylation inhibitor MLN4924 (Soucy et al., 2009) was utilized (Figures 2E and 2F). MLN4924 treatment caused the higher-molecular-weight band corresponding to NEDDylated CUL2 to collapse and led to concurrent HIF1α stabilization compared with DMSO-treated controls (Figures 2E and 2F). Under these conditions in FLAG-aGFP$_{6M}$-Halo-expressing cells, treatment with MLN4924 partially prevented the GFP-ULK1 (Figure 2E) and FAM83D-GFP (Figure 2F) degradation caused by HaloPROTAC-E. Interestingly, FAM83D-GFP levels were slightly destabilized with MLN4924 in the absence of HaloPROTAC-E (Figure 2F), suggesting that endogenous FAM83D expression is potentially regulated by a set of factors that may be dysregulated upon CUL-CRL inhibition (Xu et al., 2018). To assess whether GFP-

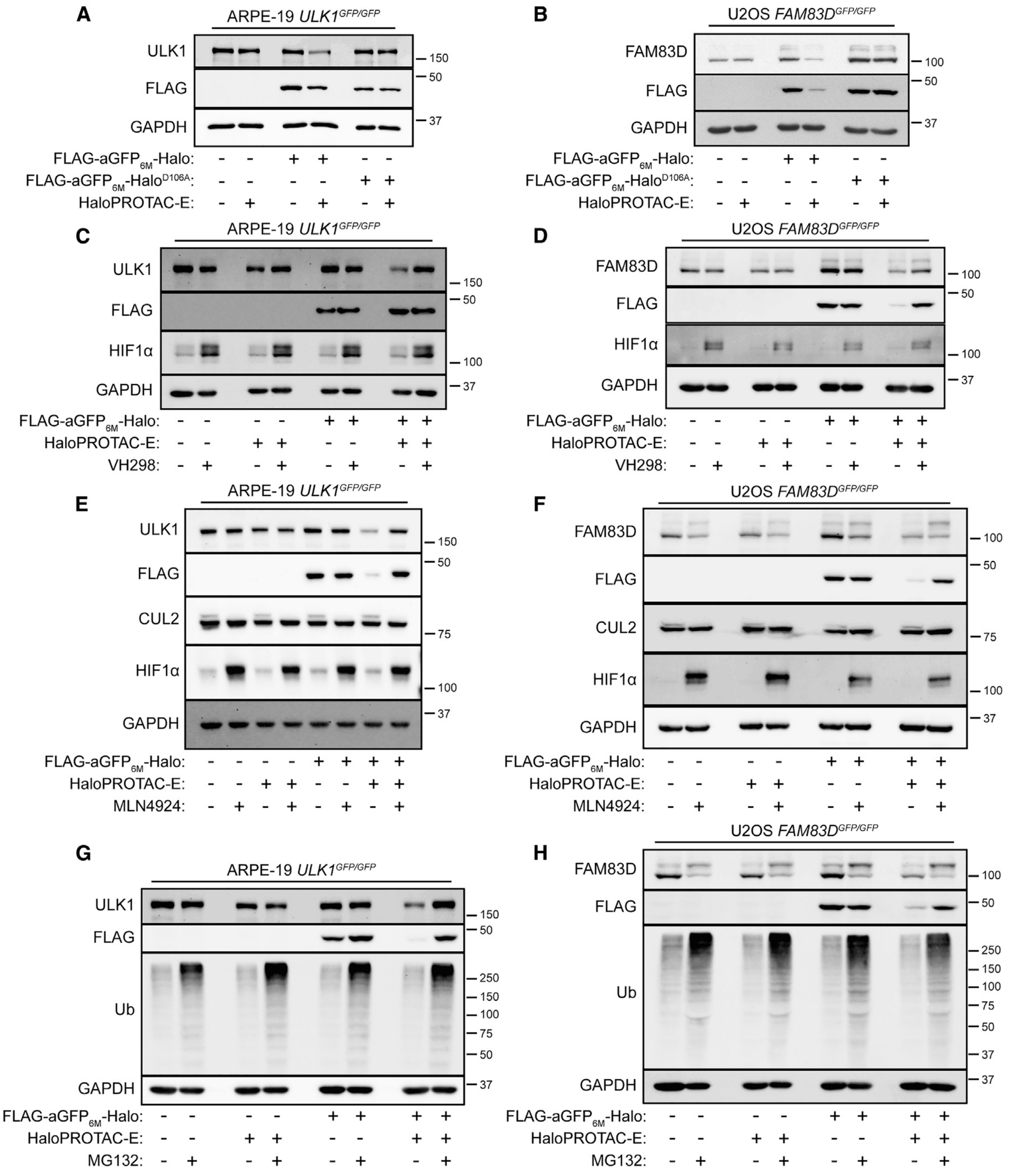

Figure 2. Characterization of HaloPROTAC-E L-AdPROM-Mediated GFP-ULK1 and FAM83D-GFP Degradation

(A and B) ARPE-19 *ULK1*$^{GFP/GFP}$ (A) or U2OS *FAM83D*$^{GFP/GFP}$ (B) FLAG-empty, FLAG-aGFP$_{6M}$-Halo and FLAG-aGFP$_{6M}$-Halo^{D106A} binding mutant-expressing cells were treated with 250 nM (A) or 1 μM (B) HaloPROTAC-E for 24 h.

(C and D) ARPE-19 *ULK1*$^{GFP/GFP}$ (C) or U2OS *FAM83D*$^{GFP/GFP}$ (D) FLAG-empty and FLAG-aGFP$_{6M}$-Halo-expressing cells were treated with 250 nM (C) or 1 μM (D) HaloPROTAC-E and 50 μM VHL inhibitor VH298 for 24 h.

(E and F) ARPE-19 *ULK1*$^{GFP/GFP}$ (E) or U2OS *FAM83D*$^{GFP/GFP}$ (F) FLAG-empty and FLAG-aGFP$_{6M}$-Halo-expressing cells were treated with 250 nM (E) or 1 μM (F) HaloPROTAC-E and 1 μM pan-Cullin NEDDylation inhibitor MLN4924 for 24 h.

(G and H) ARPE-19 *ULK1*$^{GFP/GFP}$ (G) or U2OS *FAM83D*$^{GFP/GFP}$ (H) FLAG-empty and FLAG-aGFP$_{6M}$-Halo-expressing cells were treated with 250 nM (G) or 1 μM (H) HaloPROTAC-E and 20 μM proteasome inhibitor MG132 for 24 h.

For (A)–(H), extracts were resolved by SDS-PAGE and transferred on to PVDF membranes, which were subjected to immunoblotting with indicated antibodies.

CellPress
OPEN ACCESS

ULK1 and FAM83D-GFP underwent HaloPROTAC-E-mediated proteasomal degradation, the proteasome inhibitor MG132 was utilized (Figures 2G and 2H). In FLAG-aGFP$_{6M}$-Halo-expressing cells, treatment with MG132 partially prevented the GFP-ULK1 (Figure 2G) and FAM83D-GFP (Figure 2H) degradation caused by HaloPROTAC-E. As with MLN4924 treatment, FAM83D-GFP levels were slightly destabilized with MG132 in the absence of HaloPROTAC-E (Figure 2H), suggesting that endogenous FAM83D expression is potentially regulated by a set of factors that may be dysregulated upon proteasomal inhibition.

HaloPROTAC-E L-AdPROM-Mediated Degradation of SGK3-GFP Is Comparable to that with SGK3-PROTAC1

SGK3 is a PX domain containing protein kinase that is activated at endosomes by the class 1 and 3 phosphatidylinositol 3-kinase (PI3K) family members in response to growth factors or oncogenic mutations (Bago et al., 2016; Malik et al., 2018). SGK3 is involved in the resistance to class 1 PI3K or Akt inhibitors in breast cancer as SGK3 can substitute for the loss of Akt activity and restore proliferation (Bago et al., 2016; Tovell et al., 2019b). HaloPROTAC-E was developed for the inducible degradation of SGK3, which was knocked in with a Halo-tag on one allele while another allele silenced in HEK293 cells (*SGK3*$^{Halo/-}$) (Tovell et al., 2019a). We used HEK293 SGK3 GFP KI (*SGK3*$^{GFP/GFP}$) cells (Malik et al., 2018) to test HaloPROTAC-E L-AdPROM-mediated degradation of SGK3-GFP. When *SGK3*$^{GFP/GFP}$ cell extracts expressing FLAG-empty control, FLAG-Halo-aGFP$_{6M}$, or FLAG-aGFP$_{6M}$-Halo were subjected to anti-FLAG IP, SGK3-GFP co-precipitated only with FLAG-Halo-aGFP$_{6M}$ and FLAG-aGFP$_{6M}$-Halo (Figure S4A). Treatment of *SGK3*$^{GFP/GFP}$ FLAG-empty control, FLAG-Halo-aGFP$_{6M}$ (Figure S4B), and FLAG-aGFP$_{6M}$-Halo (Figure S4C) expressing cells with increasing concentrations of HaloPROTAC-E (0.1–2 μM) for 24 h led to a reduction in SGK3-GFP levels only in cells expressing FLAG-Halo-aGFP$_{6M}$ (Figure S4B) or FLAG-aGFP$_{6M}$-Halo (Figure S4C), with optimal degradation achieved with 250 nM HaloPROTAC-E.

Next, we compared HaloPROTAC-E-mediated SGK3-Halo degradation in *SGK3*$^{Halo/-}$ cells against SGK3-GFP degradation in *SGK3*$^{GFP/GFP}$ L-AdPROM-expressing cells. HEK293 WT, *SGK3*$^{Halo/-}$, and *SGK3*$^{GFP/GFP}$ FLAG-empty, FLAG-Halo-aGFP$_{6M}$, and FLAG-aGFP$_{6M}$-Halo-expressing cells were treated with 250 nM HaloPROTAC-E for 24 h (Figures 3A and 3B). No changes in SGK3 or SGK3-GFP levels were observed in HaloPROTAC-E-treated WT or *SGK3*$^{GFP/GFP}$ cells, respectively. However, similar levels of SGK3-Halo and SGK3-GFP degradation was observed in HaloPROTAC-E-treated *SGK3*$^{Halo/-}$ and *SGK3*$^{GFP/GFP}$ cells expressing FLAG-Halo-aGFP$_{6M}$ or FLAG-aGFP$_{6M}$-Halo, respectively. To compare HaloPROTAC-E-mediated SGK3-Halo and SGK3-GFP degradation kinetics, *SGK3*$^{Halo/-}$ cells or *SGK3*$^{GFP/GFP}$ cells expressing FLAG-Halo-aGFP$_{6M}$ or FLAG-aGFP$_{6M}$-Halo were treated with 250 nM HaloPROTAC-E for 3, 6, and 24 h (Figures 3C and 3D). Although SGK3-Halo degradation was achieved slightly earlier after HaloPROTAC-E treatment, no significant difference in SGK3 levels were observed after 24 h in either *SGK3*$^{Halo/-}$ cells or L-AdPROM-expressing *SGK3*$^{GFP/GFP}$ cells. These data suggest that HaloPROTAC-E can be utilized both for the degradation of POIs knocked in with a Halo-tag or with a GFP-tag using the L-AdPROM system.

A potent SGK3-specific degrader, SGK3-PROTAC1, that binds both SGK3 and VHL was recently developed for the degradation of endogenous SGK3 (Tovell et al., 2019b). To directly compare SGK3-PROTAC1 with HaloPROTAC-E L-AdPROM-mediated SGK3 degradation, HEK293 WT and *SGK3*$^{GFP/GFP}$ FLAG-empty control, FLAG-Halo-aGFP$_{6M}$, and FLAG-aGFP$_{6M}$-Halo-expressing cells were treated with 250 nM of either SGK3-PROTAC1 or HaloPROTAC-E for 24 h (Figures 3E and 3F). As expected, no changes in SGK3 or SGK3-GFP levels were observed with HaloPROTAC-E in WT or *SGK3*$^{GFP/GFP}$ FLAG-empty cells, respectively, while degradation of both was observed with SGK3-PROTAC1. Interestingly, similar levels of SGK3-GFP degradation were observed with SGK3-PROTAC1 or HaloPROTAC-E in *SGK3*$^{GFP/GFP}$ cells expressing FLAG-Halo-aGFP$_{6M}$ or FLAG-aGFP$_{6M}$-Halo.

HaloPROTAC-E L-AdPROM-Mediated GFP-ULK1, FAM83D-GFP, and SGK3-GFP Degradation Is Reversible

We have demonstrated that following the expression of the L-AdPROM construct in cells harboring a POI-GFP, HaloPROTAC-E treatment induces robust POI-GFP degradation (Figures 1, 2, and 3). For a truly tractable system, when HaloPROTAC-E is removed, POI-GFP degradation should cease and stabilize thereafter. Therefore, we wanted to determine the reversibility of HaloPROTAC-E-mediated GFP-ULK1, FAM83D-GFP, and SGK3-GFP degradation in ARPE-19 *ULK1*$^{GFP/GFP}$ (Figures 4A and 4B), U2OS *FAM83D*$^{GFP/GFP}$ (Figures 4C and 4D), and HEK293 *SGK3*$^{GFP/GFP}$ (Figures 4E and 4F) L-AdPROM-expressing cells, respectively. Cells were treated with or without HaloPROTAC-E for 24 h, washed with PBS to remove the compound or maintained in the presence of HaloPROTAC-E, and POI-GFP levels were assessed up to 48 h thereafter. Both GFP-ULK1 (Figures 4A and 4B) and FAM83D-GFP (Figures 4C and 4D) levels were restored in a time-dependent manner reaching near control levels after 24 h, and SGK3-GFP (Figures 4E and 4F) levels after 48 h. POI-GFP degradation was sustained at all time points in cells that were maintained in HaloPROTAC-E. As expected, no changes in POI-GFP levels were observed in DMSO-treated controls following similar wash-out as HaloPROTAC-E. These data suggest that HaloPROTAC-E-mediated POI-GFP degradation through the L-AdPROM system is reversible.

HaloPROTAC-E L-AdPROM-Mediated GFP-ULK1 Degradation Inhibits Starvation-Induced Autophagy

ULK1 functions in a complex with FIP200 (focal adhesion kinase family interacting protein of 200 kDa) and ATG (autophagy-related protein) 13 (ATG13) for the regulation of autophagy initiation (Ganley et al., 2009; Jung et al., 2009; Hosokawa et al., 2009). To investigate whether the GFP-ULK1:ATG13:FIP200 interaction is affected following HaloPROTAC-E-mediated GFP-ULK1 degradation, ARPE-19 *ULK1*$^{GFP/GFP}$ FLAG-empty control and FLAG-aGFP$_{6M}$-Halo-expressing cells were treated with HaloPROTAC-E and subjected to anti-ATG13 IP (Figure 5A). Both GFP-ULK1 and FIP200 co-precipitated with ATG13 in *ULK1*$^{GFP/GFP}$ FLAG-empty and FLAG-aGFP$_{6M}$-Halo-expressing cells, suggesting that the expression of FLAG-aGFP$_{6M}$-Halo does not interfere with the interaction of GFP-ULK1

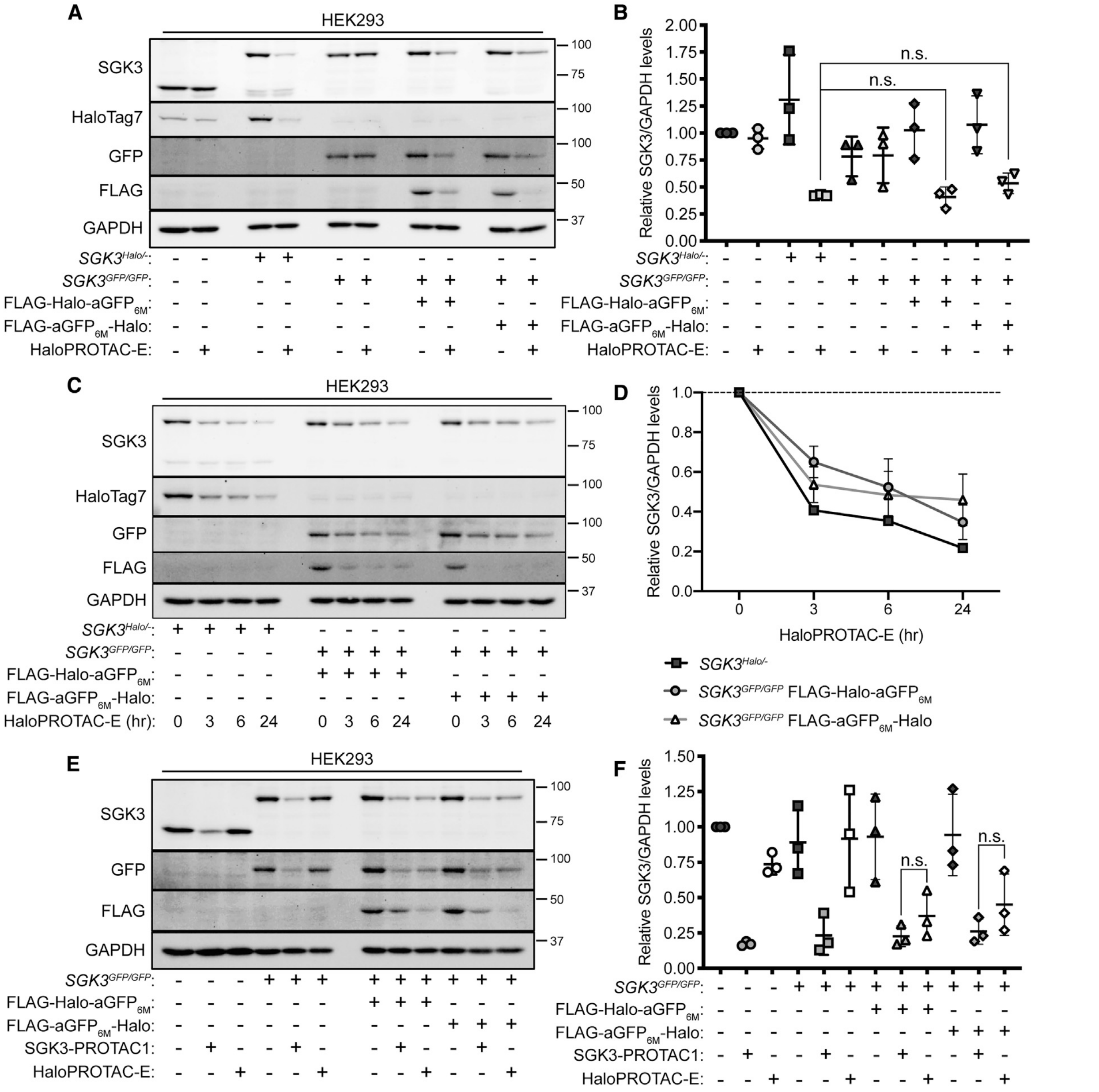

Figure 3. HaloPROTAC-E L-AdPROM-Mediated Degradation of SGK3-GFP Is Comparable to that with SGK3-PROTAC1

(A) HEK293 WT, *SGK3*$^{Halo/-}$, *SGK3*$^{GFP/GFP}$ FLAG-empty, FLAG-Halo-aGFP$_{6M}$ and FLAG-aGFP$_{6M}$-Halo-expressing cells were treated with 250 nM HaloPROTAC-E for 24 h. Extracts were resolved by SDS-PAGE and transferred on to PVDF membranes, which were subjected to immunoblotting with indicated antibodies.

(B) Quantification of relative SGK3 protein levels from (A) normalized to loading control ± SD of n = 3 independent experiments.

(C) As in (A), except *SGK3*$^{Halo/-}$ and *SGK3*$^{GFP/GFP}$ FLAG-Halo-aGFP$_{6M}$ and FLAG-aGFP$_{6M}$-Halo-expressing cells were treated with 250 nM HaloPROTAC-E for indicated times.

(D) Quantification of relative SGK3 protein levels from (C) normalized to loading control ± SD of n = 3 independent experiments.

(E) As in (A), except HEK293 WT, *SGK3*$^{GFP/GFP}$ FLAG-empty, FLAG-Halo-aGFP$_{6M}$, and FLAG-aGFP$_{6M}$-Halo-expressing cells were treated with 250 nM SGK3-PROTAC1 or HaloPROTAC-E for 24 h.

(F) Quantification of relative SGK3 protein levels from (E) normalized to loading control ± SD of n = 3 independent experiments.

Statistical analyses were carried out by one-way analysis of variance using Tukey's post-test; n.s., not significant.

with either ATG13 or FIP200. In addition, both GFP-ULK1 and FIP200 co-precipitated with ATG13 in HaloPROTAC-E-treated *ULK1*$^{GFP/GFP}$ FLAG-empty cells, suggesting that HaloPROTAC-E itself does not interfere with the GFP-ULK1:ATG13:FIP200 interaction. Following HaloPROTAC-E-mediated GFP-ULK1 degradation in FLAG-aGFP$_{6M}$-Halo cells, FIP200 but not GFP-ULK1 co-precipitated with ATG13, suggesting that ATG13 and FIP200 can still interact in the absence of GFP-ULK1, consistent with previous reports using RNAi-mediated depletion of ULK1 (Ganley et al., 2009).

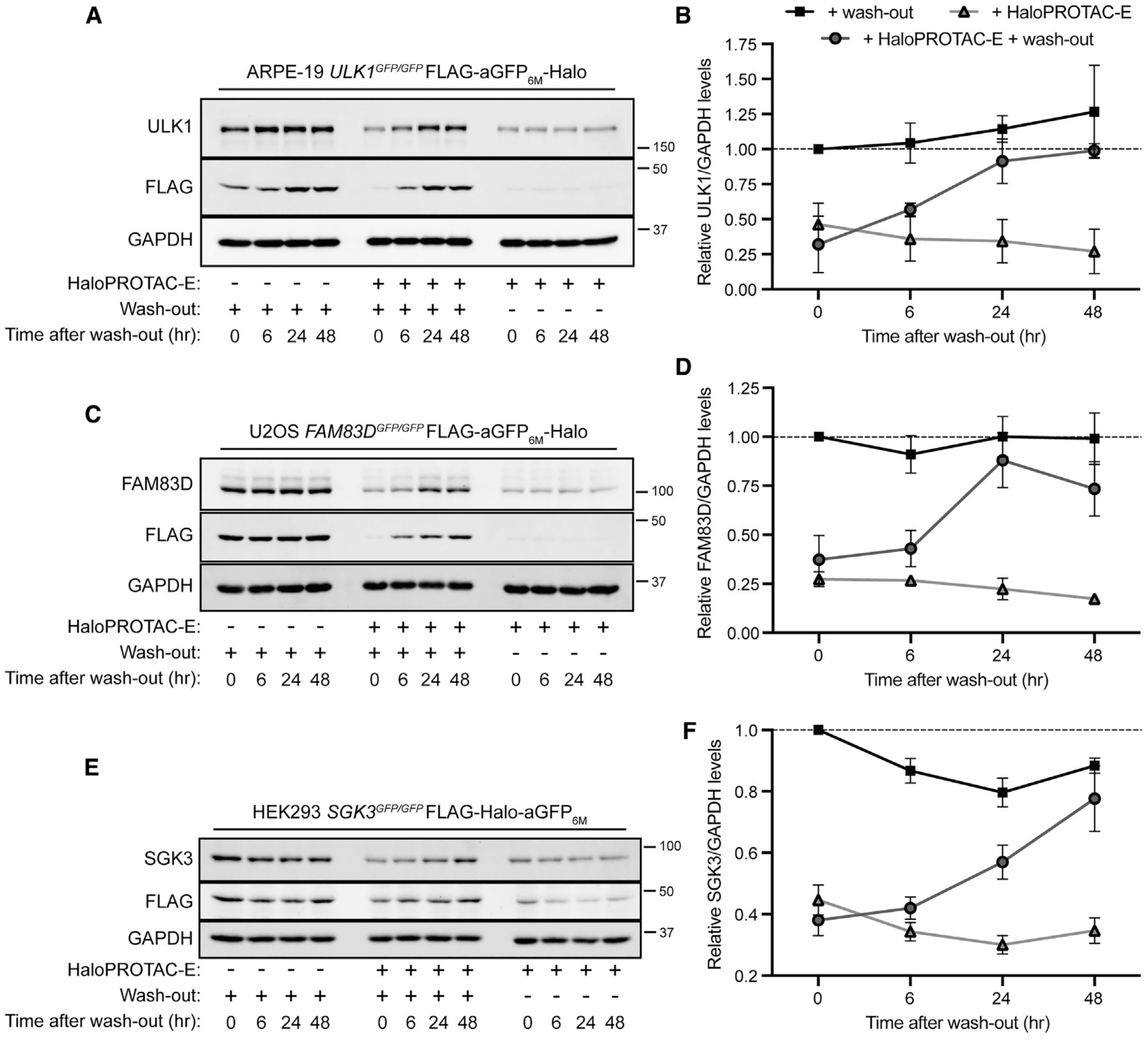

Figure 4. HaloPROTAC-E L-AdPROM-mediated GFP-ULK1, FAM83D-GFP, and SGK3-GFP Degradation Is Reversible

(A) ARPE-19 *ULK1*$^{GFP/GFP}$ FLAG-aGFP$_{6M}$-Halo-expressing cells were treated with 250 nM HaloPROTAC-E for 24 h. Cells were then either washed three times with PBS and medium replaced or maintained in the presence of HaloPROTAC-E and lysed after the indicated times. Extracts were resolved by SDS-PAGE and transferred on to PVDF membranes, which were subjected to immunoblotting with indicated antibodies.

(B) Quantification of relative GFP-ULK1 protein levels from (A) normalized to GAPDH ± SD of n = 3 independent experiments.

(C) As in (A), except U2OS *FAM83D*$^{GFP/GFP}$ FLAG-aGFP$_{6M}$-Halo-expressing cells were treated with 1 μM HaloPROTAC-E for 24 h.

(D) Quantification of relative FAM83D-GFP protein levels from (C) normalized to GAPDH ± SD of n = 3 independent experiments.

(E) As in (A), except HEK293 *SGK3*$^{GFP/GFP}$ FLAG-Halo-aGFP$_{6M}$-expressing cells were treated with 250 nM HaloPROTAC-E for 24 h.

(F) Quantification of relative SGK3-GFP protein levels from (E) normalized to GAPDH ±SD of n = 3 independent experiments.

Under nutrient-rich conditions, the mammalian target of rapamycin complex 1 (mTORC1) phosphorylates ULK1 at multiple sites, including S757, to inhibit autophagy (Kim et al., 2011). During periods of nutrient deprivation, mTORC1 is inactivated and the inhibitory phosphorylations on ULK1 are removed, resulting in increased ULK1 kinase activity (Ganley et al., 2009; Jung et al., 2009; Hosokawa et al., 2009; Kim et al., 2011). This leads to downstream autophagy signaling, including phosphorylation of ATG13 at S318 by activated ULK1 (Joo et al., 2011), expansion of the autophagosome, marked by LC3 lipidation (LC3-II), which engulfs cargo and then fuses with the lysosome for cargo degradation (Zachari and Ganley, 2017). To investigate the effects of HaloPROTAC-E L-AdPROM-mediated GFP-ULK1 degradation on downstream starvation-induced autophagy signaling, HaloPROTAC-E-treated FLAG-aGFP$_{6M}$-Halo-expressing ARPE-19 *ULK1*$^{GFP/GFP}$ cells were starved of amino acids for 2 h with Earle's balanced salt solution (EBSS) (Figure 5B). During this period, cells were also treated either with or without Bafilomycin-A1 (Baf-A1), which inhibits lysosomal degradation and prevents autophagosome clearance. The resultant accumulation of LC3-II can be used to monitor autophagic flux (Yoshimori et al., 1991; Mauvezin et al., 2015; Klionsky et al., 2016). In both EBSS-treated *ULK1*$^{GFP/GFP}$ FLAG-empty control and FLAG-aGFP$_{6M}$-Halo-expressing cells, a similar reduction in

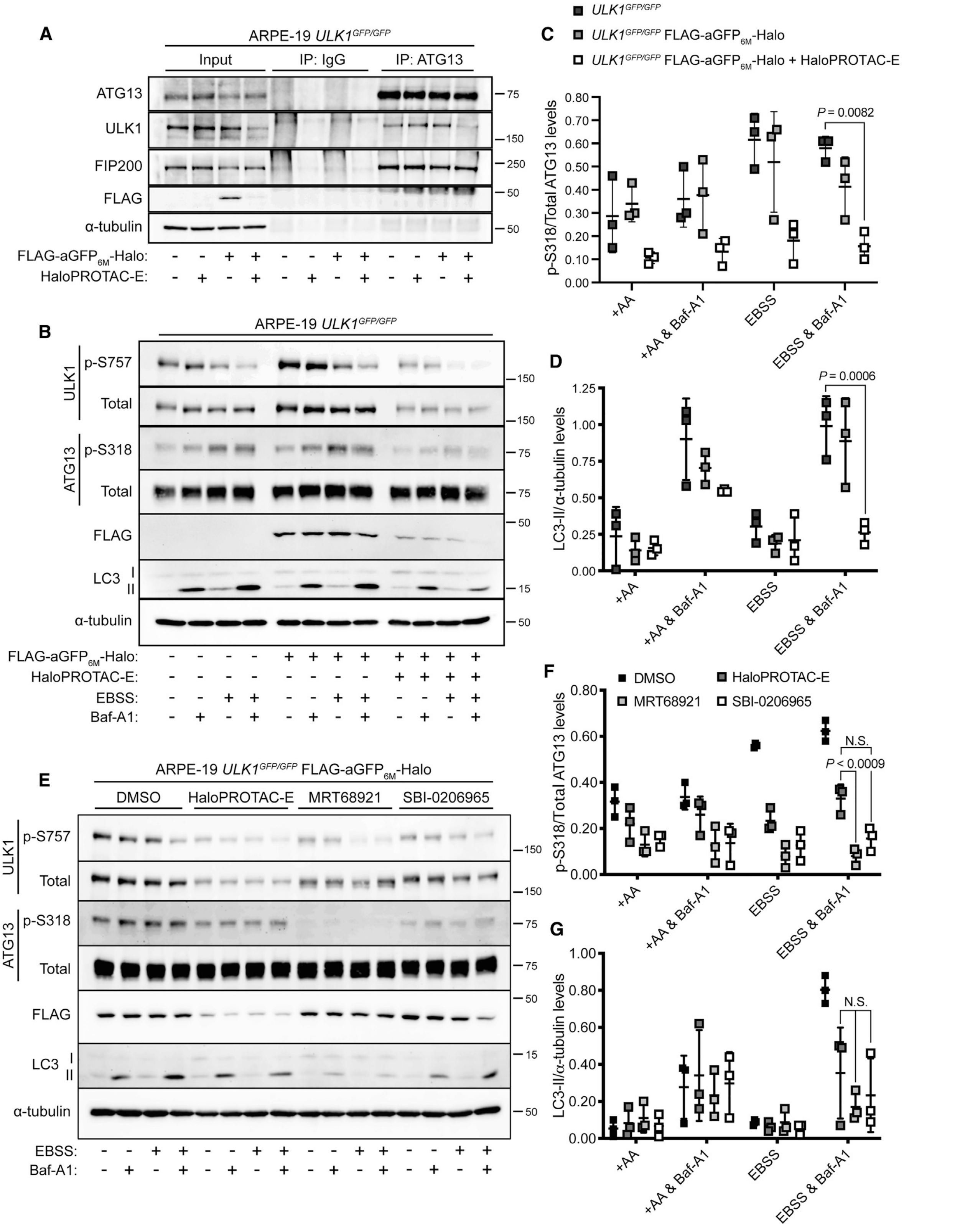

Figure 5. HaloPROTAC-E L-AdPROM-mediated GFP-ULK1 Degradation Inhibits Starvation-Induced Autophagy

(A) ARPE-19 *ULK1*$^{GFP/GFP}$ FLAG-empty and FLAG-aGFP$_{6M}$-Halo-expressing cells were treated with 250 nM HaloPROTAC-E for 24 h and subjected to ATG13 or IgG IP.

(legend continued on next page)

CellPress
OPEN ACCESS

GFP-ULK1 phosphorylation at S757 was observed (Figure 5B), indicating inhibition of mTOR. In addition, ATG13 phosphorylation at S318 also increased, demonstrating concomitant activation of GFP-ULK1 and confirming that the expression of FLAG-aGFP$_{6M}$-Halo does not interfere with GFP-ULK1 regulation during starvation-induced autophagy (Figures 5B and 5C). However, following HaloPROTAC-E-mediated GFP-ULK1 degradation, the EBSS-induced increase in ATG13 phosphorylation was attenuated (Figures 5B and 5C), demonstrating that HaloPROTAC-E L-AdPROM-mediated GFP-ULK1 degradation blocks downstream GFP-ULK1 signaling. Importantly, this results in the inhibition of starvation-induced autophagy, as indicated by the large reduction in LC3-II flux that occurs in *ULK1*$^{GFP/GFP}$ FLAG-aGFP$_{6M}$-Halo-expressing cells in the presence of HaloPROTAC-E compared with DMSO-treated and FLAG-empty controls (Figures 5B and 5D).

Next, we wanted to compare the efficacy of GFP-ULK1 degradation to inhibition by the small-molecule ULK1 inhibitors MRT68921 (Petherick et al., 2015) and SBI-0206965 (Egan et al., 2015). ARPE-19 *ULK1*$^{GFP/GFP}$ FLAG-aGFP$_{6M}$-Halo-expressing cells were pre-treated with either HaloPROTAC-E, MRT68921, or SBI-0206965, followed by EBSS and Baf-A1 for 2 h (Figure 5E). Under starvation conditions, the reduction in ATG13 phosphorylation at Ser318 relative to untreated controls was comparable between HaloPROTAC-E- and SBI-0206965-treated cells (Figures 5E and 5F). ATG13 phosphorylation was reduced further in MRT68921-treated cells (Figures 5E and 5F), potentially due to the increased potency of MRT68921 compared with SBI-0206965 (Petherick et al., 2015; Egan et al., 2015). However, under starvation conditions, LC3-II levels were comparable between HaloPROTAC-E-, MRT68921-, and SBI-0206965-treated cells (Figures 5E and 5G). These data suggest that the attenuation of starvation-induced autophagy observed following HaloPROTAC-E L-AdPROM-mediated GFP-ULK1 degradation reflects that of small-molecule inhibition.

HaloPROTAC-E L-AdPROM-Mediated FAM83D-GFP Degradation Prevents CK1α Recruitment to the Mitotic Spindle during Mitosis

Recently, we reported that FAM83D interacts with and delivers CK1α to the mitotic spindle (Fulcher et al., 2019). In both WT U2OS cells and those knocked in homozygously with both FAM83D-GFP and mCherry (mCh)-CK1α (*FAM83D*$^{GFP/GFP}$*CSNK1A1*$^{mCh/mCh}$), FAM83D was shown to direct CK1α to the mitotic spindle for proper spindle positioning and timely cell division (Fulcher et al., 2019). However in *FAM83D*-KO cells, generated using CRISPR/Cas9, CK1α is no longer recruited to the mitotic spindle, resulting in pronounced spindle positioning defects and a prolonged cell division (Fulcher et al., 2019). We sought to investigate whether HaloPROTAC-E L-AdPROM-mediated degradation of FAM83D-GFP from *FAM83D*$^{GFP/GFP}$*CSNK1A1*$^{mCh/mCh}$ cells affects recruitment of mCh-CK1α to the mitotic spindle. First, we tested whether the mitotic interaction between FAM83D-GFP and mCh-CK1α was affected following the expression of FLAG-aGFP$_{6M}$-Halo in *FAM83D*$^{GFP/GFP}$*CSNK1A1*$^{mCh/mCh}$ cells, which were synchronized in mitosis using the Eg5 inhibitor S-trityl-L-cysteine (STLC) (Fulcher et al., 2019) (Figure 6A). Anti-GFP IPs from both *FAM83D*$^{GFP/GFP}$*CSNK1A1*$^{mCh/mCh}$ FLAG-empty control and FLAG-aGFP$_{6M}$-Halo-expressing cells both co-precipitated mCh-CK1α in mitotic but not asynchronous extracts (Figure 6A), suggesting that FLAG-aGFP$_{6M}$-Halo expression alone does not interfere with the mitotic FAM83D-GFP:mCh-CK1α interaction. As predicted, anti-GFP IPs from asynchronous or mitotic WT extracts did not pull down FAM83D or CK1α. Next, when *FAM83D*$^{GFP/GFP}$*CSNK1A1*$^{mCh/mCh}$ FLAG-empty control and FLAG-aGFP$_{6M}$-Halo-expressing cells were treated with 1 μM HaloPROTAC-E for 24 h, a reduction in FAM83D-GFP levels was observed only in cells expressing FLAG-aGFP$_{6M}$-Halo (Figure 6B), while no degradation of mCh-CK1α was observed in either cell lines. To investigate the localization of FAM83D-GFP and mCh-CK1α at the mitotic spindle following HaloPROTAC-E L-AdPROM-mediated FAM83D-GFP degradation, WT and *FAM83D*$^{GFP/GFP}$*CSNK1A1*$^{mCh/mCh}$ FLAG-empty, and FLAG-aGFP$_{6M}$-Halo-expressing cells treated with HaloPROTAC-E were synchronized using STLC, fixed, and analyzed by anti-FLAG immunostaining and GFP and mCh fluorescence microscopy (Figure 6C). FAM83D-GFP, mCh-CK1α, and FLAG-aGFP$_{6M}$-Halo localized at the mitotic spindle in *FAM83D*$^{GFP/GFP}$*CSNK1A1*$^{mCh/mCh}$ cells expressing FLAG-aGFP$_{6M}$-Halo, while these mitotic localization signals were abolished with HaloPROTAC-E (Figures 6C and 6D), suggesting that the recruitment of mCh-CK1α to the mitotic spindle is inhibited by targeted degradation of FAM83D-GFP through the HaloPROTAC-E L-AdPROM system. No change in mCh-CK1α mitotic spindle localization was observed in HaloPROTAC-E-treated *FAM83D*$^{GFP/GFP}$*CSNK1A1*$^{mCh/mCh}$ FLAG-empty control cells compared with DMSO-treated controls or DMSO-treated FLAG-aGFP$_{6M}$-Halo-expressing cells (Figures 6C and 6D). These data suggest that HaloPROTAC-E alone or the expression of FLAG-aGFP$_{6M}$-Halo in U2OS *FAM83D*$^{GFP/GFP}$ *CSNK1A1*$^{mCh/mCh}$ cells does not interfere with the mitotic localization of either FAM83D-GFP or mCh-CK1α.

Untagged Endogenous RAS Proteins Are Degraded with HaloPROTAC-E in Cells Expressing FLAG-Halo-aHRAS

The proof-of-concept degradation of multiple POI-GFP KI proteins through the aGFP$_{6M}$ L-AdPROM with HaloPROTAC-E suggested that endogenous untagged POIs could be targeted

(B) ARPE-19 *ULK1*$^{GFP/GFP}$ FLAG-empty and FLAG-aGFP$_{6M}$-Halo-expressing cells were pre-treated with 250 nM HaloPROTAC-E for 24 h followed by either EBSS or 50 nM Bafilomycin-A1 (Baf-A1) for 2 h.

(C and D) Quantification of (C) p-S318 ATG13 normalized to total ATG13 protein levels and (D) LC3-II protein levels normalized to α-tubulin from (B) ± SD of n = 3 independent experiments. +AA indicates amino acid-rich conditions.

(E) ARPE-19 *ULK1*$^{GFP/GFP}$ FLAG-aGFP$_{6M}$-Halo-expressing cells were pre-treated with 250 nM HaloPROTAC-E for 24 h or with the ULK1 inhibitors MRT68921 (2 μM) or SBI-0206965 (5 μM) for 2 h followed by either EBSS or 50 nM Baf-A1 for 2 h.

(F and G) Quantification of (F) p-S318 ATG13 normalized to total ATG13 protein levels and (G) LC3-II protein levels normalized to α-tubulin from (E) ± SD of n = 3 independent experiments.

Statistical analyses were carried out by one-way analysis of variance using Tukey's post-test. For (A), (B), and (E), extracts and IPs were resolved by SDS-PAGE and transferred on to PVDF membranes, which were subjected to immunoblotting with indicated antibodies.

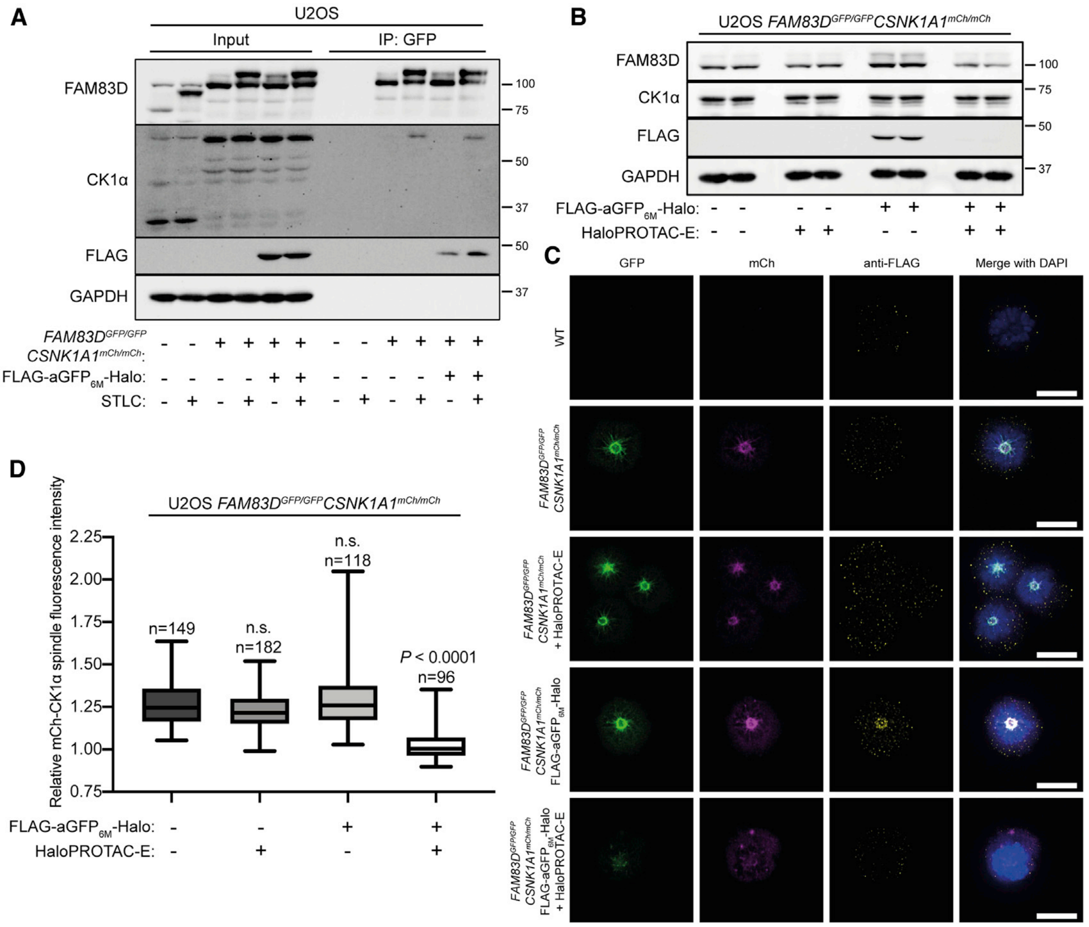

Figure 6. HaloPROTAC-E L-AdPROM-Mediated FAM83D-GFP Degradation Prevents CK1α Recruitment to the Mitotic Spindle during Mitosis
(A) U2OS WT, *FAM83D*$^{GFP/GFP}$*CSNK1A1*$^{mCh/mCh}$ FLAG-empty and FLAG-aGFP$_{6M}$-Halo-expressing cells were synchronized in mitosis using the Eg5 inhibitor S-trityl-L-cysteine (STLC) (5 μM) for 16 h. Following incubation, mitotic (M) cells were isolated through shake-off. Asynchronous (AS) cells were included as a control. Cells were washed twice with ice-cold PBS, lysed and subjected to anti-GFP IP.
(B) U2OS *FAM83D*$^{GFP/GFP}$*CSNK1A1*$^{mCh/mCh}$ FLAG-empty and FLAG-aGFP$_{6M}$-Halo-expressing cells were treated with 1 μM HaloPROTAC-E for 24 h. For (A) and (B), extracts and IPs were resolved by SDS-PAGE and transferred on to PVDF membranes, which were subjected to immunoblotting with indicated antibodies.
(C) U2OS WT, *FAM83D*$^{GFP/GFP}$*CSNK1A1*$^{mCh/mCh}$ FLAG-empty and FLAG-aGFP$_{6M}$-Halo-expressing cells were pre-treated with 1 μM HaloPROTAC-E for 24 h, synchronized in mitosis using STLC (5 μM, 16 h) and subjected to anti-FLAG immunofluorescence and GFP and mCherry (mCh) fluorescence microscopy. DNA is stained with DAPI. Scale bars, 10 μm.
(D) Quantification of mCh-CK1α spindle localization for cells described in (C). Boxplot whiskers denote the minimum and maximum measured values. The middle line represents the median, and the box ranges depict the 25th/75th percentiles.
Statistical analysis was carried out on indicated number of cells by one-way analysis of variance using Dunnett's post-test, n = 2 independent experiments; n.s., not significant.

for degradation by substituting aGFP$_{6M}$ with high-affinity binders of endogenous POIs. In this context, an anti-H-RAS monobody (aHRAS), which binds to and immunoprecipitates both H- and K-RAS, but not N-RAS, has been reported previously (Spencer-Smith et al., 2017). The RAS GTPases, including H-, K-, and N-RAS, represent the most common mediators of oncogenesis in humans (Cox et al., 2014; Hobbs et al., 2016). Specifically, 20%–50% of non-small cell lung carcinomas (NSCLC) harbor K-RAS mutations (Marabese et al., 2015; Forest et al., 2017; Jia et al., 2017). However, the therapeutic targeting of K-RAS, either by conventional pharmacological inhibition (Cox et al., 2014; Papke and Der, 2017) or targeted degradation (Zeng et al., 2020), has proven extremely challenging. Recently, we conjugated aHRAS to VHL to mediate constitutive RAS degradation following retroviral transduction in A549 NSCLC cells (Röth et al., 2019). By conjugating aHRAS to the Halo-tag and tagging with a FLAG reporter, we sought to develop an L-AdPROM system to degrade untagged endogenous RAS proteins only in the presence of HaloPROTAC-E (Figure 7A).

Following the expression of FLAG-aHRAS and FLAG-Halo-aHRAS by retroviral transduction in WT A549 cells, which harbor the K-RAS^{G12S} mutation (COSMIC cell lines project), cell extracts were subjected to anti-FLAG IP (Figure 7B). pan-RAS only co-precipitated with anti-FLAG IPs from both FLAG-aHRAS and FLAG-Halo-aHRAS-expressing cells and was

CellPress
OPEN ACCESS

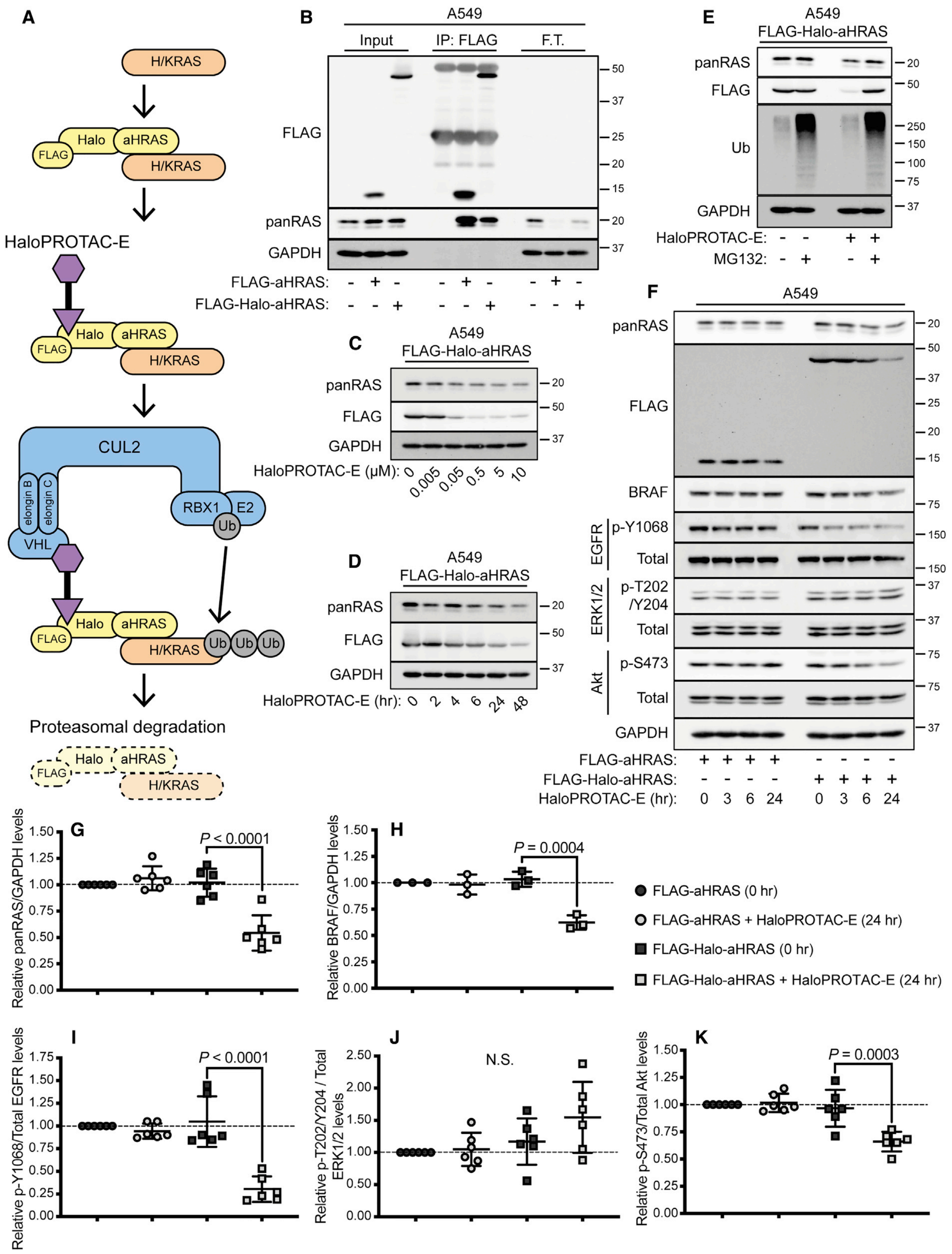

(legend on next page)

depleted from flow-through extracts, suggesting that the conjugation of aHRAS to Halo does not affect the ability of aHRAS to interact with RAS proteins. Next, to assess HaloPROTAC-E-mediated RAS degradation in FLAG-Halo-aHRAS-expressing cells, cells were treated with increasing concentrations of HaloPROTAC-E (0.005–10 μM) for 24 h (Figure 7C). A reduction in both panRAS and FLAG levels was observed with 0.5, 5, and 10 μM HaloPROTAC-E. To analyze RAS degradation kinetics in HaloPROTAC-E-treated FLAG-Halo-aHRAS-expressing cells, cells were treated with 500 nM HaloPROTAC-E for 2, 4, 6, 24, and 48 h (Figure 7D). Both panRAS and FLAG displayed time-dependent degradation upon treatment with HaloPROTAC-E in FLAG-Halo-aHRAS-expressing cells, with robust degradation achieved after 24 and 48 h. To assess whether HaloPROTAC-E L-AdPROM-mediated RAS degradation was facilitated by the proteasome, the proteasome inhibitor MG132 was utilized (Figure 7E). In FLAG-Halo-aHRAS-expressing cells, treatment with MG132 partially prevented panRAS degradation with HaloPROTAC-E, confirming that HaloPROTAC-E-mediated RAS degradation in FLAG-Halo-aHRAS-expressing cells is proteasome-dependent.

Activating K-RAS mutations are known to result in the upregulation of signaling pathways involved in tumor cell growth and survival, including the mitogen-activated protein kinase (MAPK) and PI3K/Akt signaling pathways (Affolter et al., 2013; Okudela et al., 2004; Ding et al., 2008). Following HaloPROTAC-E-mediated RAS degradation in FLAG-Halo-aHRAS-expressing cells, we analyzed the status of basal MAPK and PI3K/Akt pathway components, specifically the levels of BRAF, EGFR p-Y1068, ERK1/2 p-T202/Y204, and Akt p-S473 (Figure 7F). Relative to untreated control cells, no significant changes in levels of panRAS, BRAF, EGFR p-Y1068, ERK1/2 p-T202/Y204, or Akt p-S473 were observed in FLAG-aHRAS-expressing cells after 24 h with HaloPROTAC-E (Figures 7F–7K), suggesting that HaloPROTAC-E alone does not affect MAPK or PI3K/Akt signaling. However, under these conditions, significant reduction in panRAS, BRAF, EGFR p-Y1068, and Akt p-S473 levels were observed in FLAG-Halo-aHRAS-expressing cells treated with HaloPROTAC-E for 24 h, although ERK1/2 p-T202/Y204 levels did not change significantly (Figures 7F–7K). These data suggest that HaloPROTAC-E-mediated RAS degradation in FLAG-Halo-aHRAS expressing A549 cells appears to reduce RAS-driven PI3K/Akt signaling downstream.

DISCUSSION

In this report, we have combined the use of polypeptide binders of specific POIs with a HaloPROTAC to engineer a tractable L-AdPROM system for inducible degradation of POIs. We utilized L-AdPROM to mediate the inducible degradation of endogenously GFP-tagged ULK1, FAM83D, and SGK3 in ARPE-19, U2OS, and HEK293 cells, respectively, after treatment with a HaloPROTAC. While degradation of target proteins was not complete, this seems to be a limitation of targeted proteolysis in general, as SGK3 was degraded to a similar degree with both HaloPROTAC L-AdPROM and SGK3-PROTAC1. Crucially, the level of degradation achieved for GFP-ULK1 using the HaloPROTAC L-AdPROM system was sufficient to inhibit the function of GFP-ULK1 in the initiation of starvation-induced autophagy. Similarly, FAM83D-GFP degradation led to the inhibition of CK1α recruitment to the mitotic spindle. However, it is important to note that, as with any RNA/protein knockdown approach, the impact on specific POI biological function may vary depending on the level of POI degradation achieved.

HaloPROTACs are already being used for the degradation of POIs where the Halo-tag is introduced with CRISPR/Cas9 genome editing (Tovell et al., 2019a), similar to how GFP-tags were introduced on our targets. Our HaloPROTAC L-AdPROM system offers an alternative to tagging POIs with Halo for targeted POI degradation. In addition, for any existing POI-GFP KI cell lines, which are routinely generated for immunofluorescence and proteomics applications, or for those POIs for which polypeptide binders exist, our L-AdPROM system serves as a readymade tool for dissecting the biological consequences of POI degradation.

The level of SGK3-GFP degradation achieved using the HaloPROTAC L-AdPROM system was similar to that achieved with a potent SGK3-specific PROTAC. In principle, the L-AdPROM system can thus be exploited not only to explore the biological role of the POI but also rapidly validate the phenotypic effects of UPS-mediated POI degradation, before the development of more resource-intensive POI-specific PROTACs. It is important to note that the ubiquitylation sites on POIs caused by POI-specific PROTACs and L-AdPROM are likely to be different, but the information on the phenotypic consequences resulting from the levels of POI degradation achieved is nonetheless valuable. The principle for recruitment of a promiscuous E3 ligase to ubiquitylate POIs is at the heart of both PROTACs and L-AdPROM, and as long as POIs are degraded by the UPS, determining which

Figure 7. Untagged Endogenous RAS Proteins Are Degraded with HaloPROTAC-E in Cells Expressing FLAG-Halo-aHRAS

(A) Schematic representation of FLAG-Halo-aHRAS HaloPROTAC L-AdPROM system.

(B) A549 FLAG-empty, FLAG-aHRAS, and FLAG-Halo-aHRAS-expressing cells were lysed and subjected to IP with anti-FLAG M2 resin. F.T., post-IP flow-through extract.

(C) A549 FLAG-Halo-aHRAS-expressing cells were treated with increasing concentrations of HaloPROTAC-E (0–10 μM) for 24 h.

(D) A549 FLAG-Halo-aHRAS-expressing cells were treated with 500 nM HaloPROTAC-E for indicated times (0–48 h).

(E) A549 FLAG-Halo-aHRAS-expressing cells were treated with 500 nM HaloPROTAC-E and 20 μM proteasome inhibitor MG132 for 24 h.

(F) A549 FLAG-aHRAS and FLAG-Halo-aHRAS-expressing cells were treated with 500 nM HaloPROTAC-E for indicated times (0, 3, 6, and 24 h).

For (B–F), extracts and IPs were resolved by SDS-PAGE and transferred on to PVDF membranes, which were subjected to immunoblotting with indicated antibodies.

(G–K) Quantification from (F) of relative (G) panRAS normalized to GAPDH protein levels (n = 6 ± SD), (H) BRAF normalized to GAPDH protein levels (n = 3 ± SD), (I) p-Y1068 EGFR normalized to total EGFR protein levels (n = 6 ± SD), (J) p-T202/Y204 ERK1/2 normalized to total ERK1/2 protein levels (n = 6 ± SD), and (K) p-S473 Akt normalized to total Akt protein levels (n = 6 ± SD) in the absence or presence of HaloPROTAC-E (500 nM, 24 h).

Statistical analyses were carried out by one-way analysis of variance using Tukey's post-test.

CellPress
OPEN ACCESS

lysine residues on the POIs are ubiquitylated in each system does not necessarily inform a uniform mode of action. However, in certain cases with tagged POIs, the ubiquitylation of the tag itself can be sufficient to cause the fused POI to be degraded, as was recently exemplified (Zeng et al., 2020).

The L-AdPROM has two crucial interlinked components: the Halo-tag, which binds the HaloPROTAC, and the POI-specific polypeptide binder, which binds the target POI. In principle, the polypeptide binder can be substituted for any high-affinity POI-specific binder and the resulting L-AdPROM system should target the POI for degradation only upon treatment of cells with the HaloPROTAC. In theory, this approach could then be applied to any cell systems without the need for CRISPR/Cas9 genome editing to introduce GFP- or Halo-tags on POIs. For proof-of-principle, we substituted the anti-GFP nanobody for a monobody that specifically binds H- and K-RAS and achieved degradation of unmodified endogenous RAS proteins only in the presence of the HaloPROTAC. The level of RAS degradation achieved in the presence of Halo-PROTAC-E in FLAG-Halo-aHRAS expressing A549 cells was sufficient to reduce RAS-driven signaling downstream. Ideally, the development of high-affinity polypeptide binders with a higher degree of specificity, for example, a K-RAS^{G12C}-specific binder, needs to be explored to further expand the capability of this approach.

The benefits of PROTAC technologies over conventional small-molecule inhibitors, such as the capability of PROTACs to specifically reduce target protein levels at nanomolar concentrations (Bondeson and Crews, 2017; Lucas and Ciulli, 2017) as well as eliminating the scaffolding role of the protein (Burslem et al., 2018), can be harnessed with our L-AdPROM system. Currently the optimal inhibition of ULK1 using specifically designed small-molecule inhibitors still requires compound concentrations ranging from 1 to 10 μM (Petherick et al., 2015; Egan et al., 2015; Martin et al., 2018; Zachari et al., 2020). We observed that the attenuation in starvation-induced autophagy following GFP-ULK1 degradation with 250 nM HaloPROTAC-E reflects that of GFP-ULK1 inhibition using MRT68921 or SBI-0206965. As well as ULK1, MRT68921 (Petherick et al., 2015) and SBI-0206965 (Martin et al., 2018; Dite et al., 2018) have been reported to also inhibit a number of additional kinases. Therefore, the employment of a targeted protein degradation approach, such as the L-Ad-PROM system, can potentially overcome off-target effects observed with conventional pharmacological inhibitors, in addition to eliminating the potential scaffolding roles the protein kinases may also perform.

One concern with regard to the utilization of the L-AdPROM system is that the introduction and expression of a 48-kDa complex might negatively interfere with the biological function of the POI. Although we observe slight POI-GFP stabilization following the expression of FLAG-aGFP$_{6M}$-Halo, expression in ARPE-19 *ULK1$^{GFP/GFP}$* or U2OS *FAM83D$^{GFP/GFP}$CSNK1A1$^{mCh/mCh}$* cells did not appear to interfere with GFP-ULK1 or FAM83D-GFP functions, respectively. Nonetheless, potential impact on POI function needs to be evaluated on a case-by-case basis. Following the expression of FLAG-aGFP$_{6M}$-Halo in U2OS *FAM83D$^{GFP/GFP}$CSNK1A1$^{mCh/mCh}$* cells, no stabilization of mCh-CK1α was observed, nor were there any changes in mCh-CK1α levels with HaloPROTAC-E, while FAM83D-GFP was robustly degraded. To fully ascertain potential off-target effects of the L-AdPROM system, a quantitative proteomics approach could be used to determine the potential changes in stability of other proteins following the expression of FLAG-aGFP$_{6M}$-Halo and/or with HaloPROTAC-E.

The L-AdPROM system presented here further expands on the currently available targeted protein degradation technologies that can be exploited, each possessing their own benefits and limitations (Roth et al., 2019). The auxin-inducible degron (AID) system, for example, achieves rapid degradation of POIs either knocked in with the AID (Natsume et al., 2016) or of GFP-tagged POIs following the expression of AID fused to an anti-GFP nanobody (Daniel et al., 2018) in the presence of indole-3-acetic acid (IAA). However, for use in mammalian cells, the AID system also requires the overexpression of the plant-based F box transport inhibitor response 1 (TIR1) protein. Furthermore, relatively large IAA concentrations of 500 μM are required to induce POI degradation in cells (Natsume et al., 2016; Daniel et al., 2018), which has been reported to be toxic at high concentrations due to IAA oxidation by eukaryotic peroxidases (Folkes et al., 1999). In contrast, no effect on cell viability was reported by MTS assay in WT HEK293 cells treated with 0.001–1 μM HaloPROTAC-E for 48 h (Tovell et al., 2019a). In addition, the dTAG system (Nabet et al., 2018) exploited a stable FKBP12 mutant, FKBP12^{F36V}, which contains a ligand-binding cavity, to develop a FKBP12^{F36V}-CRBN-based PROTAC, dTAG-13. Using CRISPR/Cas9 to tag a POI with FKBP12^{F36V} (POI-FKBP12^{F36V}), POI-FKBP12^{F36V} degradation through the CUL4-CRL machinery was observed in the presence of dTAG-13 at nanomolar concentrations. FKBP12^{F36V} is a smaller tag than Halo and uses a non-covalent ligand. Therefore, an L-Ad-PROM system which substitutes Halo for FKBP12^{F36V}, conjugated to a high-affinity small polypeptide binder, may prove a viable option for dTAG-13-mediated POI degradation, where the homozygous integration of a non-fluorescent FKBP12^{F36V}-tag using CRISPR/Cas9 is not feasible.

SIGNIFICANCE

A ligand-inducible affinity-directed protein missile (L-Ad-PROM) technology for tractable and reversible degradation of desired intracellular proteins of interest (POIs) is described. We demonstrate that targeted POI degradation using the L-AdPROM system leads to loss of protein function. The L-AdPROM technology is versatile and adaptable, where, in principle, the small polypeptide binder can be substituted for any high-affinity POI-targeting binder. Therefore, this technology offers an excellent opportunity for any researcher wishing to dissect the function of potentially any intracellular POI. Targeted degradation of POIs potentially overcomes the key limitations of CRISPR/Cas9-mediated gene knockouts, which are irreversible and not possible when targeting essential genes, as well as RNA interference approaches, which often require prolonged treatments and are commonly associated with off-target effects. Our technology can be exploited to rapidly inform the utility of UPS-mediated POI degradation before the resource-intensive and lengthy development of POI-specific PROTACs.

STAR★METHODS

Detailed methods are provided in the online version of this paper and include the following:

- KEY RESOURCES TABLE
- RESOURCE AVAILABILITY
  - Lead Contact
  - Materials Availability
  - Data and Code Availability
- EXPERIMENTAL MODEL AND SUBJECT DETAILS
  - Cell Lines
- METHOD DETAILS
  - Plasmids
  - Generation of Cell Lines Using CRISPR/Cas9
  - Retroviral Generation of Stable Cell Lines
  - Treatment of Cells with Compounds
  - Cell Lysis and Immunoprecipitation
  - SDS-PAGE and Western Blotting
  - Immunofluorescence Microscopy
- QUANTIFICATION AND STATISTICAL ANALYSIS

SUPPLEMENTAL INFORMATION

Supplemental Information can be found online at https://doi.org/10.1016/j.chembiol.2020.06.013.

ACKNOWLEDGMENTS

This study was funded by the UK Medical Research Council (MRC), awarded to G.P.S. (MC_UU_00018/6 and MC_UU_12016/3). G.P.S. and I.G.G. are also supported by the pharmaceutical companies supporting the Division of Signal Transduction Therapy (Boehringer-Ingelheim, GlaxoSmithKline, Merck-Serono). L.M.S. is supported by the UK MRC Prize PhD studentship. A.C. is funded by the European Research Council (ERC) under the European Union's Seventh Framework Program as a Starting Grant (ERC-2012-StG-311460 DrugE3CRLs) and receives or has received sponsored research support from Boehringer Ingelheim, Eisai Inc., Nurix Inc., Ono Pharma, and Amphista Therapeutics. We thank members of the G.P.S., I.G.G., and A.C. labs for their highly appreciated experimental advice and discussions during the course of these experiments. We thank the Alessi Lab, MRC PPU, University of Dundee, for kindly providing HEK293 *SGK3*$^{GFP/GFP}$ and *SGK3*$^{Halo/-}$ cells. We thank E. Allen, L. Fin, J. Stark, and A. Muir for assistance with tissue culture, the staff at the DNA Sequencing services (School of Life Sciences, University of Dundee) and the cloning, antibody, and protein production teams within the MRC PPU Reagents & Services (University of Dundee) coordinated by J. Hastie. We thank the staff at the Dundee Imaging Facility (School of Life Sciences, University of Dundee) for their invaluable advice throughout this project.

AUTHOR CONTRIBUTIONS

L.M.S. performed most experiments, collected and analyzed the data, and contributed to the writing of the manuscript. T.J.M. designed strategies and developed methods for all of the CRISPR/Cas9 gene editing and AdPROM constructs used in this study. A.N. generated and characterized ARPE-19 *ULK1*$^{GFP/GFP}$ cells. A.T. and C.M. designed and synthesized compounds. L.J.F., S.R., A.C., and I.G.G. provided critical inputs on experiments through the course of this study and contributed to the writing of the manuscript. G.P.S. conceived the project, analyzed data, and contributed to the writing of the manuscript.

DECLARATION OF INTERESTS

A.C. is a scientific founder, non-executive director, consultant and shareholder of Amphista Therapeutics, a company that is developing targeted protein degradation therapeutic platforms.

Received: March 5, 2020
Revised: May 19, 2020
Accepted: June 19, 2020
Published: July 14, 2020

REFERENCES

Affolter, A., Drigotas, M., Fruth, K., Schmidtmann, I., Brochhausen, C., Mann, W.J., and Brieger, J. (2013). Increased radioresistance via G12S K-Ras by compensatory upregulation of MAPK and PI3K pathways in epithelial cancer. Head Neck *35*, 220–228.

Allan, C., Burel, J.M., Moore, J., Blackburn, C., Linkert, M., Loynton, S., Macdonald, D., Moore, W.J., Neves, C., Patterson, A., Porter, M., Tarkowska, A., Loranger, B., and Avondo, J. (2012). OMERO: flexible, model-driven data management for experimental biology. Nat Methods *9*, 245–253.

Bago, R., Sommer, E., Castel, P., Crafter, C., Bailey, F.P., Shpiro, N., Baselga, J., Cross, D., Eyers, P.A., and Alessi, D.R. (2016). The hVps34-SGK3 pathway alleviates sustained PI3K/Akt inhibition by stimulating mTORC1 and tumour growth. EMBO J. *35*, 1902–1922.

Bondeson, D.P., and Crews, C.M. (2017). Targeted protein degradation by small molecules. Annu. Rev. Pharmacol. Toxicol. *57*, 107–123.

Bondeson, D.P., Mares, A., Smith, I.E., Ko, E., Campos, S., Miah, A.H., Mulholland, K.E., Routly, N., Buckley, D.L., Gustafson, J.L., et al. (2015). Catalytic in vivo protein knockdown by small-molecule PROTACs. Nat. Chem. Biol. *11*, 611–617.

Bozatzi, P., and Sapkota, G.P. (2018). The FAM83 family of proteins: from pseudo-PLDs to anchors for CK1 isoforms. Biochem. Soc. Trans. *46*, 761–771.

Buckley, D.L., Raina, K., Darricarrere, N., Hines, J., Gustafson, J.L., Smith, I.E., Miah, A.H., Harling, J.D., and Crews, C.M. (2015). HaloPROTACS: use of small molecule PROTACs to induce degradation of halotag fusion proteins. ACS Chem. Biol. *10*, 1831–1837.

Burslem, G.M., Smith, B.E., Lai, A.C., Jaime-Figueroa, S., Mcquaid, D.C., Bondeson, D.P., Toure, M., Dong, H., Qian, Y., Wang, J., et al. (2018). The advantages of targeted protein degradation over inhibition: an RTK case study. Cell Chem. Biol. *25*, 67–77 e3.

Cardote, T.A.F., Gadd, M.S., and Ciulli, A. (2017). Crystal structure of the Cul2-Rbx1-EloBC-VHL ubiquitin ligase complex. Structure *25*, 901–911.e3.

Cong, L., Ran, F.A., Cox, D., Lin, S., Barretto, R., Habib, N., Hsu, P.D., Wu, X., Jiang, W., Marraffini, L.A., and Zhang, F. (2013). Multiplex genome engineering using CRISPR/Cas systems. Science *339*, 819–823.

Cox, A.D., Fesik, S.W., Kimmelman, A.C., LUO, J., and DER, C.J. (2014). Drugging the undruggable RAS: mission possible? Nat. Rev. Drug Discov. *13*, 828–851.

Daniel, K., Icha, J., Horenburg, C., Muller, D., Norden, C., and Mansfeld, J. (2018). Conditional control of fluorescent protein degradation by an auxin-dependent nanobody. Nat. Commun. *9*, 3297.

Ding, L., Getz, G., Wheeler, D.A., Mardis, E.R., Mclellan, M.D., Cibulskis, K., Sougnez, C., Greulich, H., Muzny, D.M., Morgan, M.B., et al. (2008). Somatic mutations affect key pathways in lung adenocarcinoma. Nature *455*, 1069–1075.

Dite, T.A., Langendorf, C.G., Hoque, A., Galic, S., Rebello, R.J., Ovens, A.J., Lindqvist, L.M., Ngoei, K.R.W., Ling, N.X.Y., Furic, L., et al. (2018). AMP-activated protein kinase selectively inhibited by the type II inhibitor SBI-0206965. J. Biol. Chem. *293*, 8874–8885.

Doudna, J.A., and Charpentier, E. (2014). Genome editing. The new frontier of genome engineering with CRISPR-Cas9. Science *346*, 1258096.

Douglass, E.F.,, JR., Miller, C.J., Sparer, G., Shapiro, H., and Spiegel, D.A. (2013). A comprehensive mathematical model for three-body binding equilibria. J. Am. Chem. Soc. *135*, 6092–6099.

Egan, D.F., Chun, M.G., Vamos, M., Zou, H., Rong, J., Miller, C.J., Lou, H.J., Raveendra-Panickar, D., Yang, C.C., Sheffler, D.J., et al. (2015). Small molecule inhibition of the autophagy kinase ULK1 and identification of ULK1 substrates. Mol. Cell *59*, 285–297.

Elbashir, S.M., Harborth, J., Lendeckel, W., Yalcin, A., Weber, K., and Tuschl, T. (2001). Duplexes of 21-nucleotide RNAs mediate RNA interference in cultured mammalian cells. Nature *411*, 494–498.

Folkes, L.K., Dennis, M.F., Stratford, M.R., Candeias, L.P., and Wardman, P. (1999). Peroxidase-catalyzed effects of indole-3-acetic acid and analogues on lipid membranes, DNA, and mammalian cells in vitro. Biochem. Pharmacol. *57*, 375–382.

Forest, F., Stachowicz, M.L., Casteillo, F., Karpathiou, G., Gouzy-Grosjean, F., Guilaubey, C., Cottier, M., Beal, J., Clemenson, A., and Peoc'h, M. (2017). EGFR, KRAS, BRAF and HER2 testing in metastatic lung adenocarcinoma: value of testing on samples with poor specimen adequacy and analysis of discrepancies. Exp. Mol. Pathol. *103*, 306–310.

Frost, J., Galdeano, C., Soares, P., Gadd, M.S., Grzes, K.M., Ellis, L., Epemolu, O., Shimamura, S., Bantscheff, M., Grandi, P., et al. (2016). Potent and selective chemical probe of hypoxic signalling downstream of HIF-alpha hydroxylation via VHL inhibition. Nat. Commun. *7*, 13312.

Fulcher, L.J., Bozatzi, P., Tachie-Menson, T., Wu, K.Z.L., Cummins, T.D., Bufton, J.C., Pinkas, D.M., Dunbar, K., Shrestha, S., Wood, N.T., et al. (2018). The DUF1669 domain of FAM83 family proteins anchor casein kinase 1 isoforms. Sci. Signal. *11*, eaao2341.

Fulcher, L.J., He, Z., Mei, L., Macartney, T.J., Wood, N.T., Prescott, A.R., Whigham, A.J., Varghese, J., Gourlay, R., Ball, G., et al. (2019). FAM83D directs protein kinase CK1alpha to the mitotic spindle for proper spindle positioning. EMBO Rep. *20*, e47495.

Fulcher, L.J., Hutchinson, L.D., Macartney, T.J., Turnbull, C., and Sapkota, G.P. (2017). Targeting endogenous proteins for degradation through the affinity-directed protein missile system. Open Biol. *7*, 170066.

Fulcher, L.J., Macartney, T., Bozatzi, P., Hornberger, A., Rojas-Fernandez, A., and Sapkota, G.P. (2016). An affinity-directed protein missile system for targeted proteolysis. Open Biol. *6*, 160255.

Gadd, M.S., Testa, A., Lucas, X., Chan, K.H., Chen, W., Lamont, D.J., Zengerle, M., and Ciulli, A. (2017). Structural basis of PROTAC cooperative recognition for selective protein degradation. Nat. Chem. Biol. *13*, 514–521.

Ganley, I.G., Lam Du, H., Wang, J., Ding, X., Chen, S., and Jiang, X. (2009). ULK1.ATG13.FIP200 complex mediates mTOR signaling and is essential for autophagy. J. Biol. Chem. *284*, 12297–12305.

Hobbs, G.A., Der, C.J., and Rossman, K.L. (2016). RAS isoforms and mutations in cancer at a glance. J. Cell Sci. *129*, 1287–1292.

Hosokawa, N., Hara, T., Kaizuka, T., Kishi, C., Takamura, A., Miura, Y., Iemura, S., Natsume, T., Takehana, K., Yamada, N., et al. (2009). Nutrient-dependent mTORC1 association with the ULK1-Atg13-FIP200 complex required for autophagy. Mol. Biol. Cell *20*, 1981–1991.

Ivan, M., Kondo, K., Yang, H., Kim, W., Valiando, J., Ohh, M., Salic, A., Asara, J.M., Lane, W.S., and Kaelin, W.G.,, JR. (2001). HIFalpha targeted for VHL-mediated destruction by proline hydroxylation: implications for O_2 sensing. Science *292*, 464–468.

Jaakkola, P., Mole, D.R., Tian, Y.M., Wilson, M.I., Gielbert, J., Gaskell, S.J., Von Kriegsheim, A., Hebestreit, H.F., Mukherji, M., Schofield, C.J., et al. (2001). Targeting of HIF-alpha to the von Hippel-Lindau ubiquitylation complex by O_2-regulated prolyl hydroxylation. Science *292*, 468–472.

Jansen, L.E., Black, B.E., Foltz, D.R., and Cleveland, D.W. (2007). Propagation of centromeric chromatin requires exit from mitosis. J. Cell Biol. *176*, 795–805.

Jia, Y., Jiang, T., Li, X., Zhao, C., Zhang, L., Zhao, S., Liu, X., Qiao, M., Luo, J., Shi, J., et al. (2017). Characterization of distinct types of KRAS mutation and its impact on first-line platinum-based chemotherapy in Chinese patients with advanced non-small cell lung cancer. Oncol. Lett. *14*, 6525–6532.

Joo, J.H., Dorsey, F.C., Joshi, A., Hennessy-Walters, K.M., Rose, K.L., Mccastlain, K., Zhang, J., Iyengar, R., Jung, C.H., Suen, D.F., et al. (2011). Hsp90-Cdc37 chaperone complex regulates Ulk1- and Atg13-mediated mitophagy. Mol. Cell *43*, 572–585.

Jung, C.H., Jun, C.B., Ro, S.H., Kim, Y.M., Otto, N.M., Cao, J., Kundu, M., and Kim, D.H. (2009). ULK-Atg13-FIP200 complexes mediate mTOR signaling to the autophagy machinery. Mol. Biol. Cell *20*, 1992–2003.

Kim, J., Kundu, M., Viollet, B., and Guan, K.L. (2011). AMPK and mTOR regulate autophagy through direct phosphorylation of Ulk1. Nat. Cell Biol. *13*, 132–141.

Klionsky, D.J., Abdelmohsen, K., Abe, A., Abedin, M.J., Abeliovich, H., Acevedo Arozena, A., Adachi, H., Adams, C.M., Adams, P.D., Adeli, K., et al. (2016). Guidelines for the use and interpretation of assays for monitoring autophagy (Third Edition). Autophagy *12*, 1–222.

Los, G.V., Encell, L.P., Mcdougall, M.G., Hartzell, D.D., Karassina, N., Zimprich, C., Wood, M.G., Learish, R., Ohana, R.F., Urh, M., et al. (2008). HaloTag: a novel protein labeling technology for cell imaging and protein analysis. ACS Chem. Biol. *3*, 373–382.

Lucas, X., and Ciulli, A. (2017). Recognition of substrate degrons by E3 ubiquitin ligases and modulation by small-molecule mimicry strategies. Curr. Opin. Struct. Biol. *44*, 101–110.

Malik, N., Macartney, T., Hornberger, A., Anderson, K.E., Tovell, H., Prescott, A.R., and Alessi, D.R. (2018). Mechanism of activation of SGK3 by growth factors via the Class 1 and Class 3 PI3Ks. Biochem. J. *475*, 117–135.

Marabese, M., Ganzinelli, M., Garassino, M.C., Shepherd, F.A., Piva, S., Caiola, E., Macerelli, M., Bettini, A., Lauricella, C., Floriani, I., et al. (2015). KRAS mutations affect prognosis of non-small-cell lung cancer patients treated with first-line platinum containing chemotherapy. Oncotarget *6*, 34014–34022.

Martin, K.R., Celano, S.L., Solitro, A.R., Gunaydin, H., Scott, M., O'hagan, R.C., Shumway, S.D., Fuller, P., and Mackeigan, J.P. (2018). A potent and selective ULK1 inhibitor suppresses autophagy and sensitizes cancer cells to nutrient stress. iScience *8*, 74–84.

Mauvezin, C., Nagy, P., Juhasz, G., and Neufeld, T.P. (2015). Autophagosome-lysosome fusion is independent of V-ATPase-mediated acidification. Nat. Commun. *6*, 7007.

Nabet, B., Roberts, J.M., Buckley, D.L., Paulk, J., Dastjerdi, S., Yang, A., Leggett, A.L., Erb, M.A., Lawlor, M.A., Souza, A., et al. (2018). The dTAG system for immediate and target-specific protein degradation. Nat. Chem. Biol. *14*, 431–441.

Natsume, T., Kiyomitsu, T., Saga, Y., and Kanemaki, M.T. (2016). Rapid protein depletion in human cells by auxin-inducible degron tagging with short homology donors. Cell Rep. *15*, 210–218.

Neklesa, T.K., Tae, H.S., Schneekloth, A.R., Stulberg, M.J., Corson, T.W., Sundberg, T.B., Raina, K., Holley, S.A., and Crews, C.M. (2011). Small-molecule hydrophobic tagging-induced degradation of HaloTag fusion proteins. Nat. Chem. Biol. *7*, 538–543.

Ohana, R.F., Encell, L.P., Zhao, K., Simpson, D., Slater, M.R., Urh, M., and Wood, K.V. (2009). HaloTag7: a genetically engineered tag that enhances bacterial expression of soluble proteins and improves protein purification. Protein Expr. Purif. *68*, 110–120.

Ohh, M., Park, C.W., Ivan, M., Hoffman, M.A., Kim, T.Y., Huang, L.E., Pavletich, N., Chau, V., and Kaelin, W.G. (2000). Ubiquitination of hypoxia-inducible factor requires direct binding to the beta-domain of the von Hippel-Lindau protein. Nat. Cell Biol. *2*, 423–427.

Okudela, K., Hayashi, H., Ito, T., Yazawa, T., Suzuki, T., Nakane, Y., Sato, H., Ishi, H., Keqin, X., Masuda, A., et al. (2004). K-ras gene mutation enhances motility of immortalized airway cells and lung adenocarcinoma cells via Akt activation: possible contribution to non-invasive expansion of lung adenocarcinoma. Am. J. Pathol. *164*, 91–100.

Papke, B., and Der, C.J. (2017). Drugging RAS: know the enemy. Science *355*, 1158–1163.

Petherick, K.J., Conway, O.J., Mpamhanga, C., Osborne, S.A., Kamal, A., Saxty, B., and Ganley, I.G. (2015). Pharmacological inhibition of ULK1 kinase blocks mammalian target of rapamycin (mTOR)-dependent autophagy. J. Biol. Chem. *290*, 11376–11383.

Pines, J., and Lindon, C. (2005). Proteolysis: anytime, any place, anywhere? Nat. Cell Biol. *7*, 731–735.

Roos-Mattjus, P., and Sistonen, L. (2004). The ubiquitin-proteasome pathway. Ann. Med. *36*, 285–295.

Rossi, A., Kontarakis, Z., Gerri, C., Nolte, H., Holper, S., Kruger, M., and Stainier, D.Y. (2015). Genetic compensation induced by deleterious mutations but not gene knockdowns. Nature *524*, 230–233.

Roth, S., Fulcher, L.J., and Sapkota, G.P. (2019). Advances in targeted degradation of endogenous proteins. Cell Mol. Life Sci. *76*, 2761–2777.

Röth, S., Macartney, T.J., Konopacka, A., Queisser, M.A., and Sapkota, G.P. (2019). Targeting endogenous K-RAS for degradation through the affinity-directed protein missile system. bioRxiv, 805150.

Sakamoto, K.M., Kim, K.B., Kumagai, A., Mercurio, F., Crews, C.M., and Deshaies, R.J. (2001). Protacs: chimeric molecules that target proteins to the Skp1-Cullin-F box complex for ubiquitination and degradation. Proc. Natl. Acad. Sci. U S A *98*, 8554–8559.

Sander, J.D., and Joung, J.K. (2014). CRISPR-Cas systems for editing, regulating and targeting genomes. Nat. Biotechnol. *32*, 347–355.

Scheffner, M., Nuber, U., and Huibregtse, J.M. (1995). Protein ubiquitination involving an E1-E2-E3 enzyme ubiquitin thioester cascade. Nature *373*, 81–83.

Schneider, C.A., Rasband, W.S., and Eliceiri, K.W. (2012). NIH Image to ImageJ: 25 years of image analysis. Nat. Methods *9*, 671–675.

Smoak, E.M., Stein, P., Schultz, R.M., Lampson, M.A., and Black, B.E. (2016). Long-term retention of CENP-A nucleosomes in mammalian oocytes underpins transgenerational inheritance of centromere identity. Curr. Biol. *26*, 1110–1116.

Soucy, T.A., Smith, P.G., Milhollen, M.A., Berger, A.J., Gavin, J.M., Adhikari, S., Brownell, J.E., Burke, K.E., Cardin, D.P., Critchley, S., et al. (2009). An inhibitor of NEDD8-activating enzyme as a new approach to treat cancer. Nature *458*, 732–736.

Spencer-Smith, R., Koide, A., Zhou, Y., Eguchi, R.R., Sha, F., Gajwani, P., Santana, D., Gupta, A., Jacobs, M., Herrero-Garcia, E., et al. (2017). Inhibition of RAS function through targeting an allosteric regulatory site. Nat. Chem. Biol. *13*, 62–68.

Tang, J.C., Drokhlyansky, E., Etemad, B., Rudolph, S., Guo, B., Wang, S., Ellis, E.G., Li, J.Z., and Cepko, C.L. (2016). Detection and manipulation of live antigen-expressing cells using conditionally stable nanobodies. eLife *5*, https://doi.org/10.7554/eLife.15312.

Tomoshige, S., Hashimoto, Y., and Ishikawa, M. (2016). Efficient protein knockdown of HaloTag-fused proteins using hybrid molecules consisting of IAP antagonist and HaloTag ligand. Bioorg. Med. Chem. *24*, 3144–3148.

Toure, M., and Crews, C.M. (2016). Small-molecule PROTACS: new approaches to protein degradation. Angew. Chem. Int. Ed. *55*, 1966–1973.

Tovell, H., Testa, A., Maniaci, C., Zhou, H., Prescott, A.R., Macartney, T., Ciulli, A., and Alessi, D.R. (2019a). Rapid and reversible knockdown of endogenously tagged endosomal proteins via an optimized HaloPROTAC degrader. ACS Chem. Biol. *14*, 882–892.

Tovell, H., Testa, A., Zhou, H., Shpiro, N., Crafter, C., Ciulli, A., and Alessi, D.R. (2019b). Design and characterization of SGK3-PROTAC1, an isoform specific SGK3 kinase PROTAC degrader. ACS Chem. Biol. *14*, 2024–2034.

Wang, T., Birsoy, K., Hughes, N.W., Krupczak, K.M., Post, Y., Wei, J.J., Lander, E.S., and Sabatini, D.M. (2015). Identification and characterization of essential genes in the human genome. Science *350*, 1096–1101.

Wenzel, D.M., Stoll, K.E., and Klevit, R.E. (2011). E2s: structurally economical and functionally replete. Biochem. J. *433*, 31–42.

Xu, B., Deng, Y., Bi, R., Guo, H., Shu, C., Shah, N.K., Chang, J., Liu, G., Du, Y., Wei, W., and Wang, C. (2018). A first-in-class inhibitor, MLN4924 (pevonedistat), induces cell-cycle arrest, senescence, and apoptosis in human renal cell carcinoma by suppressing UBE2M-dependent neddylation modification. Cancer Chemother. Pharmacol. *81*, 1083–1093.

Yoshimori, T., Yamamoto, A., Moriyama, Y., Futai, M., and Tashiro, Y. (1991). Bafilomycin A1, a specific inhibitor of vacuolar-type H(+)-ATPase, inhibits acidification and protein degradation in lysosomes of cultured cells. J. Biol. Chem. *266*, 17707–17712.

Zachari, M., and Ganley, I.G. (2017). The mammalian ULK1 complex and autophagy initiation. Essays Biochem. *61*, 585–596.

Zachari, M., Rainard, J.M., Pandarakalam, G.C., Robinson, L., Gillespie, J., Rajamanickam, M., Hamon, V., Morrison, A., Ganley, I.G., and Mcelroy, S.P. (2020). The Identification and characterisation of autophagy inhibitors from the published kinase inhibitor sets. Biochem. J. *477*, 801–814.

Zeng, M., Xiong, Y., Safaee, N., Nowak, R.P., Donovan, K.A., Yuan, C.J., Nabet, B., Gero, T.W., Feru, F., Li, L., et al. (2020). Exploring targeted degradation strategy for oncogenic KRAS(G12C). Cell Chem. Biol. *27*, 19–31 e6.

Zengerle, M., Chan, K.H., and Ciulli, A. (2015). Selective small molecule induced degradation of the BET bromodomain protein BRD4. ACS Chem. Biol. *10*, 1770–1777.

Zhao, B., Bhuripanyo, K., Schneider, J., Zhang, K., Schindelin, H., Boone, D., and Yin, J. (2012). Specificity of the E1-E2-E3 enzymatic cascade for ubiquitin C-terminal sequences identified by phage display. ACS Chem. Biol. *7*, 2027–2035.

STAR★METHODS

KEY RESOURCES TABLE

REAGENT or RESOURCE	SOURCE	IDENTIFIER
Antibodies		
Rabbit polyclonal anti-Akt	Cell Signaling Technology	Cat# 9272, RRID:AB_329827
Mouse monoclonal anti-Akt p-S473	Cell Signaling Technology	Cat# 12694, RRID:AB_2797994
Rabbit polyclonal anti-ATG13	Sigma-Aldrich	Cat# SAB4200100, RRID:AB_10602787
Sheep polyclonal anti-ATG13	MRC PPU Reagents & Services	Cat# S777C
Rabbit polyclonal anti-ATG13 p-S318	Novus	Cat# NBP2-19127
Rabbit monoclonal anti-BRAF	Thermo Fisher Scientific	Cat# 702187, RRID:AB_2633065
Rabbit polyclonal anti-CK1α	Bethyl	Cat# A301-991A RRID:AB_1576501
Sheep polyclonal anti-CK1α	MRC PPU Reagents & Services	Cat# SA527
Rabbit polyclonal anti-CUL2	Invitrogen	Cat# 51-1800, RRID:AB_2533898
Rabbit polyclonal anti-EGFR	Santa Cruz Biotechnology	Cat# sc-03, RRID:AB_631420
Rabbit monoclonal anti-EGFR p-Y1068	Cell Signaling Technology	Cat# 3777, RRID:AB_2096270
Rabbit polyclonal anti- ERK1/2	Cell Signaling Technology	Cat# 9102, RRID:AB_330744
Mouse monoclonal anti-ERK1/2 p-T202/Y204	Cell Signaling Technology	Cat# 9106, RRID:AB_331768
Sheep polyclonal anti-FAM83D	MRC PPU Reagents & Services	Cat# SA102
Rabbit polyclonal anti-FIP200	Proteintech	Cat# 17250-1-AP, RRID:AB_10666428
Mouse monoclonal HRP-conjugated anti-FLAG	Sigma-Aldrich	Cat# A8592, RRID:AB_439702
Mouse monoclonal anti-FLAG	Sigma-Aldrich	Cat# F1804, RRID:AB_262044
Rabbit monoclonal anti-GAPDH	Cell Signaling Technology	Cat# 2118, RRID:AB_561053
Sheep polyclonal anti-GFP	MRC PPU Reagents & Services	Cat# S268B
Rabbit polyclonal anti-HaloTag	Promega	Cat# G9281, RRID:AB_713650
Mouse monoclonal anti-HIF1α	BD Biosciences	Cat# 610959, RRID:AB_398272
Sheep polyclonal anti-LC3	MRC PPU Reagents & Services	Cat# S400D
Rabbit monoclonal pan-RAS	Abcam	Cat# ab206969
Sheep polyclonal anti-SGK3	MRC PPU Reagents & Services	Cat# S848D
Rat monoclonal anti-α-tubulin	Thermo Fisher Scientific	Cat# MA1-80189, RRID:AB_2210200
Mouse monoclonal anti-mono- and poly-ubiquitinylated Conjugates	Enzo Life Sciences	Cat# BML-PW8810, RRID:AB_10541840
Rabbit monoclonal anti-ULK1	Cell Signaling Technology	Cat# 8054, RRID:AB_11178668
Rabbit polyclonal anti-ULK1 p-S757	Cell Signaling Technology	Cat# 6888, RRID:AB_10829226
Goat anti-rabbit IgG HRP-conjugated	Cell Signaling Technology	Cat# 7074, RRID:AB_2099233
Rabbit anti-sheep IgG HRP-conjugated	Thermo Fisher Scientific	Cat# 31480, RRID:AB_228457
Goat anti-rat IgG HRP-conjugated	Thermo Fisher Scientific	Cat# 62-9520, RRID:AB_2533965
Goat anti-mouse IgG HRP-conjugated	Thermo Fisher Scientific	Cat# 31430, RRID:AB_228307
Goat-anti-mouse IgG Alexa-Fluor 647	Thermo Fisher Scientific	Cat# A-21235
Chemicals, Peptides, and Recombinant Proteins		
HaloPROTAC-E	Tovell et al. 2019a	N/A
SGK3-PROTAC1	Tovell et al. 2019b	N/A
VH298	Frost et al. 2016	N/A
MLN4924	Active Biochem	Cat# A-1139
MG132	Abcam	Cat# Ab141003
Bafilomycin-A1	Enzo Life Sciences	Cat# BML-CM110
MRT68921	MRC PPU Reagents & Services	N/A

(Continued on next page)

Continued		
REAGENT or RESOURCE	SOURCE	IDENTIFIER
SBI-0206965	Sigma-Aldrich	Cat# SML1540
S-trityl-L-cysteine (STLC)	Sigma-Aldrich	Cat# 164739
PEI MAX – Transfection Grade Linear PEI Hydrochloride MW 40,000	Polysciences	Cat# 24765
Polybrene (Hexadimethrine bromide)	Sigma-Aldrich	Cat# 107689
GFP-Trap-Agarose	Chromotek	Cat# GTA-20
Anti-FLAG M2 Affinity Gel	Sigma-Aldrich	Cat# A2220
Immobilon Western Chemiluminescent HRP Substrate	Merck	Cat# WBKLS0500
ProLong™ Gold Antifade Mountant with DAPI	Life Technologies	Cat# P36935
Deposited Data		
Data obtained in this study	This paper	https://data.mendeley.com/datasets/xjnf2sr577/draft?a=4dd608f3-a50c-42af-bd0f-2d470e6b0ef0
Experimental Models: Cell Lines		
A549	ATCC	Cat# CCL-185
ARPE-19	ATCC	Cat# CRL-2302
ARPE-19 *ULK1*$^{GFP/GFP}$	This paper	N/A
HEK293	ATCC	Cat# CRL-1573
HEK293 *SGK3*$^{Halo/-}$	Tovell et al. 2019a	N/A
HEK293 *SGK3*$^{GFP/GFP}$	Malik et al. 2018	N/A
HEK293-FT	Invitrogen	Cat# R70007
U2OS	ATCC	Cat# HTB-96
U2OS *FAM83D*$^{GFP/GFP}$	Fulcher et al. 2019	N/A
U2OS *FAM83D*$^{GFP/GFP}$*CSNK1A1*$^{mCh/mCh}$	Fulcher et al. 2019	N/A
Recombinant DNA		
pCMV-gag-pol	Cell Biolabs	Cat# RV-111
pCMV-VSV-G	Cell Biolabs	Cat# RV-110
pBabeD-puromycin FLAG-Halo-aGFP$_{6M}$	This paper; MRC PPU Reagents & Services	Cat# DU57764
pBabeD-puromycin FLAG-aGFP$_{6M}$-Halo	This paper; MRC PPU Reagents & Services	Cat# DU57765
pBabeD-puromycin FLAG-aGFP$_{6M}$-Halo^{D106A}	This paper; MRC PPU Reagents & Services	Cat# DU60748
pBabeD-puromycin FLAG-aHRAS	MRC PPU Reagents & Services	Cat# DU57190
pBabeD-puromycin FLAG-Halo-aHRAS	This paper; MRC PPU Reagents & Services	Cat# DU57462
pBabeD-puromycin GFP	MRC PPU Reagents & Services	Cat# DU32961
pBabeD-puromycin U6 ULK1 N-terminal knockin (KI) Sense guide RNA (gRNA)	This paper; MRC PPU Reagents & Services	Cat# DU57396
pX335 ULK1 N-terminal knockin (KI) Antisense guide RNA (gRNA) + Cas9n	This paper; MRC PPU Reagents & Services	Cat# DU57403
pMA-RQ ULK1 N-terminal GFP donor	This paper; MRC PPU Reagents & Services	Cat# DU57856
Software and Algorithms		
ImageJ	Schneider et al. 2012	https://imagej.nih.gov/ij/
SoftWoRx	GE Healthcare	N/A
OMERO	Allan et al., 2012	http://openmicroscopy.org/
Graphpad Prism v8	GraphPad Prism Inc	https://www.graphpad.com/scientific-software/prism/
CK1a spindle localisation quantification macro	Fulcher et al. 2019	N/A

RESOURCE AVAILABILITY

Lead Contact

Further information and requests for resources and reagents should be directed to and will be fulfilled by the Lead Contact, Gopal Sapkota (g.sapkota@dundee.ac.uk).

Materials Availability

All constructs used in this study are available to request from the MRC PPU Reagents & Services webpage (http://mrcppureagents.dundee.ac.uk) and the unique identifier (DU) numbers provide direct links to the cloning strategies and sequence details. All constructs were sequence-verified by the DNA Sequencing Service, University of Dundee (http://www.dnaseq.co.uk).

Data and Code Availability

The datasets generated during this study are available at Mendeley Data https://data.mendeley.com/datasets/xjnf2sr577/draft?a=4dd608f3-a50c-42af-bd0f-2d470e6b0ef0.

EXPERIMENTAL MODEL AND SUBJECT DETAILS

Cell Lines

All procedures were carried out under aseptic conditions meeting biological safety requirements. A549 cells (ATCC, Cat# CCL-185) are human epithelial lung carcinoma cells derived from a 58-year-old Caucasian male. ARPE-19 cells (ATCC, Cat# CRL-2302) are human retinal pigment epithelial cells derived from a 19-year-old male. HEK293 cells (ATCC, Cat# CRL-1573) are human embryonic kidney cells. HEK293-FT cells (Invitrogen, Cat# R70007) are a clonal isolate of HEK293 cells transformed with the SV40 large T antigen. U2OS cells (ATCC, Cat# HTB-96) are human epithelial bone osteosarcoma cells derived from a 15-year-old Caucasian female. For growth, A549, HEK293, HEK293-FT and U2OS cells were maintained in DMEM (Life Technologies) containing 10% (v/v) foetal bovine serum (FBS, Thermo Fisher Scientific), 2 mM L-glutamine (Lonza), 100 U/ml penicillin (Lonza) and 0.1 mg/ml streptomycin (Lonza). ARPE-19 cells were maintained in a 1:1 mix of DMEM and Ham's F-12 nutrient mix (Life Technologies) containing 15% (v/v) FBS, 2 mM L-glutamine, 100 U/ml penicillin and 0.1 mg/ml streptomycin. Cells were grown at 37°C with 5% CO_2 in a water-saturated incubator. For passaging, cells were incubated with trypsin/EDTA at 37°C to detach cells. For transient transfections, cells were transfected for 24 hr with indicated concentration of cDNA (per 10 ml media) in serum free Opti-MEM (Gibco) with the transfection reagent polyethylenimine (PEI; diluted in 25 mM HEPES pH 7.5).

METHOD DETAILS

Plasmids

For transient expression or production of retroviral vectors, the following were cloned into pBABED-puromycin plasmids: FLAG-Halo-aGFP$_{6M}$ (DU57764), FLAG-aGFP$_{6M}$-Halo (DU57765), FLAG-aGFP$_{6M}$-Halo^{D106A} (DU60748), FLAG-aHRAS (DU57190), FLAG-Halo-aHRAS (DU57462), GFP (DU32961). For the generation of ARPE-19 *ULK1*$^{GFP/GFP}$ cells, the following guide RNAs (gRNA) and donor constructs were generated: sense gRNA (DU57396), antisense gRNA (DU57403), GFP donor (DU57856). All constructs were sequence-verified by the DNA Sequencing Service, University of Dundee (http://www.dnaseq.co.uk). These constructs are available to request from the MRC PPU Reagents and Services webpage (http://mrcppureagents.dundee.ac.uk) and the unique identifier (DU) numbers provide direct links to the cloning strategies and sequence details.

Generation of Cell Lines Using CRISPR/Cas9

The CRISPR/Cas9 genome editing system (Cong et al., 2013) was used to generate U2OS *FAM83D* homozygous C-terminal GFP knockin (KI) (*FAM83D*$^{GFP/GFP}$) cells (Fulcher et al., 2019), U2OS *FAM83D* homozygous C-terminal GFP KI and *CSNK1A1* homozygous N-terminal mCherry (mCh) KI (*FAM83D*$^{GFP/GFP}$*CSNK1A1*$^{mCh/mCh}$) cells (Fulcher et al., 2019), HEK293 *SGK3* homozygous C-terminal GFP KI (*SGK3*$^{GFP/GFP}$) cells (Malik et al., 2018), HEK293 heterozygous *SGK3* C-terminal Halo KI (*SGK3*$^{Halo/-}$) cells (Tovell et al., 2019a), and ARPE-19 *ULK1* homozygous N-terminal GFP KI (*ULK1*$^{GFP/GFP}$) cells. For the generation of ARPE-19 *ULK1*$^{GFP/GFP}$ cells, cells were transfected with vectors encoding a pair of guide RNAs (pBABED-puromycin-sgRNA1 and pX335-CAS9-D10A-sgRNA2) targeting ULK1 exon 1 (1 mg each), along with the respective donor plasmids carrying the GFP KI insert (3 mg) and PEI. 16 hr post-transfection, selection with 2 μg/ml puromycin (Sigma-Aldrich) was carried out and continued for a further 48 hr. The transfection process was repeated one more time. After selection, cells were sorted by flow cytometry and single GFP-positive cell clones were plated on individual wells of two 96-well plates. Viable clones were expanded, and integration of GFP at the target locus was verified by Western blotting and genomic sequencing of the targeted locus.

Retroviral Generation of Stable Cell Lines

Retroviral pBABED-puromycin vectors encoding the desired construct (6 μg) were co-transfected with pCMV5-gag-pol (3.2 μg) and pCMV5-VSV-G (2.8 μg) (Cell Biolabs) into a 10 cm diameter dish of ~70% confluent HEK293-FT cells. Briefly, plasmids were added to 1 ml Opti-MEM medium to which 24 μl of 1 mg/ml PEI was added. Following a gentle mix and incubation at room temperature for

20 min, the transfection mix was added dropwise to HEK293-FT cells. 16 hr post-transfection, fresh medium was added to the cells. 24 hr later, the retroviral medium was collected and passed through 0.45 μm sterile syringe filters. Target cells (~60% confluent) were transduced with the optimised titre of the retroviral medium diluted in fresh medium (typically 1:1-1:10) containing 8 μg/ml polybrene (Sigma-Aldrich) for 24 hr. The retroviral medium was then replaced with fresh medium, and 24 hr later, the medium was again replaced with fresh medium containing 2 μg/ml puromycin for selection of cells which had integrated the constructs. A pool of transduced cells were utilised for subsequent experiments following complete death of non-transduced cells placed under selection in parallel.

Treatment of Cells with Compounds

HaloPROTAC-E, SGK3-PROTAC1 and VH298 were synthesised as previously described (Tovell et al., 2019a; Tovell et al., 2019b; Frost et al., 2016) and used at indicated concentrations and times. The following chemicals were added to cell media at indicated concentrations and times: MLN4924 (Active Biochem), MG132 (Abcam), Bafilomycin-A1 (Enzo Life Sciences), MRT68921 (MRC PPU Reagents and Services), SBI-0206965 (Sigma-Aldrich). Cells were synchronised in mitosis using the Eg5 inhibitor S-trityl-L-cysteine (STLC, Sigma-Aldrich, 5 μM, 16 hr) (Fulcher et al., 2019). Following incubation, mitotic cells were isolated through shake-off. For amino acid starvation, cells were washed twice in Earle's balanced salt solution (EBSS, Gibco) and incubated in EBSS for 2 hr.

Cell Lysis and Immunoprecipitation

Cells were harvested by washing twice with phosphate-buffered saline (PBS) and scraping into ice-cold lysis buffer (50 mM Tris-HCl pH 7.5, 0.27 M sucrose, 150 mM NaCl, 1 mM EGTA, 1 mM EDTA, 1 mM sodium orthovanadate, 10 mM sodium β-glycerophosphate, 50 mM sodium fluoride, 5 mM sodium pyrophosphate and 1% NP-40) supplemented with 1x cOmplete™ protease inhibitor cocktail (Roche). After incubation for 10 min on ice, lysates were clarified by centrifugation at 20,000 xg for 20 min at 4°C. Protein concentration was determined according to the Bradford assay to enable normalisation between samples.

Following determination of protein concentration by Bradford assay, immunoprecipitation (IP) was utilised to isolate a particular protein of interest. For anti-FLAG IPs, anti-FLAG M2 resin (Sigma-Aldrich) was used; for anti-ATG13 IPs, anti-ATG13 antibody (MRC PPU Reagents & Services, S777C) was used; for anti-GFP IPs, GFP-TRAP beads (ChromoTek) were used. Before an IP was performed, an input from each lysate was retained to compare and determine IP efficiency. Samples were incubated for 4 hr at 4°C on a rotating wheel. Beads were collected by centrifugation at 1000 xg for 1 min at 4°C and a sample of the supernatant was retained (flow-through). IPs were subsequently washed three times with lysis buffer. Input, immunoprecipitation and flow-through samples were reduced in 2x LDS sample buffer (Invitrogen).

SDS-PAGE and Western Blotting

Cell lysates containing equal amounts of protein (10-20 μg) were resolved by SDS-PAGE and transferred to PVDF membrane. Membranes were blocked in 5% (w/v) non-fat milk (Marvel) in TBS-T (50 mM Tris–HCl pH 7.5, 150 mM NaCl, 0.2% Tween-20) and incubated overnight at 4°C in 5% (w/v) BSA/TBS-T or 5% milk/TBS-T with the appropriate primary antibodies. Primary antibodies used at indicated dilutions include: anti-Akt (9272S, CST, 1:1,000), anti-Akt p-S473 (12694, CST, 1:1,000), anti-ATG13 (SAB4200100, Sigma-Aldrich, 1:1,000), anti-ATG13 p-S318 (NBP2-19127, Novus, 1:1,000), anti-BRAF (702187, Thermo Fisher Scientific, 1:1,000), anti-CK1α (A301-991A, Bethyl, 1:1,000; SA527, MRC PPU Reagents & Services, 1:1,000), anti-CUL2 (51-1800, Invitrogen, 1:1,000), anti-EGFR (sc-03, Santa Cruz, 1:1,000), anti-EGFR p-Y1068 (3777, CST, 1:1,000), anti-ERK1/2 (9102, CST, 1:1,000), anti-ERK1/2 p-T202/Y204 (9106, CST, 1:1,000), anti-FAM83D (SA102, MRC PPU Reagents & Services, 1:1,000), anti-FIP200 (17250-1-AP, Proteintech, 1:1,000), anti-FLAG (A8592, Sigma-Aldrich, 1:2,500), anti-GAPDH (2118, CST, 1:5,000), anti-GFP (S268B, MRC PPU Reagents & Services, 1:2,000), anti-HaloTag7 (Promega, G9281, 1:1,000), anti-HIF1α (610959, BD, 1:1,000), anti-LC3 (S400D, MRC PPU Reagents & Services, 1:200), anti-panRAS (ab206969, Abcam, 1:500), anti-SGK3 (S848D, MRC PPU Reagents & Services, 1:1,000), anti-α-tubulin (MA1-80189, Thermo Fisher Scientific, 1:5,000), anti-mono- and poly-ubiquitinylated conjugates (BML-PW8810, Enzo, 1:2,000), anti-ULK1 (8054, CST, 1:1,000), anti-ULK1 p-S757 (6888, CST, 1:1,000).

Membranes were subsequently washed with TBS-T and incubated with HRP-conjugated secondary antibody for 1 hr at room temperature. HRP-coupled secondary antibodies used at indicated dilutions include: goat anti-rabbit-IgG (7074, CST, 1:2,500), rabbit anti-sheep-IgG (31480, Thermo Fisher Scientific, 1:5,000), goat anti-rat IgG (62-9520, Thermo Fisher Scientific, 1:5,000), goat anti-mouse-IgG (31430, Thermo Fisher Scientific, 1:5,000). After further washing, signal detection was performed using ECL (Merck) and ChemiDoc MP System (Bio-Rad). ImageJ v1.49 (National Institutes of Health) was used to analyse protein bands by densitometry (Schneider et al., 2012).

Immunofluorescence Microscopy

Cells were seeded onto sterile glass coverslips in 6-well dishes. Coverslips were washed twice with PBS, fixed with 4% (w/v) paraformaldehyde (Thermo Fisher Scientific) for 10 min, washed twice with and incubated for 10 min in DMEM/10 mM HEPES pH 7.4. After one wash in PBS, cell permeabilisation was carried out using 0.2% NP-40 in PBS for 4 min. Samples were blocked by washing twice and incubation for 15 min in blocking buffer (1% (w/v) BSA/PBS). Coverslips were incubated for 1 hr at 37°C with primary antibodies in blocking buffer and washed three times in blocking buffer. Mouse monoclonal anti-FLAG primary antibody (F1804, Sigma-Aldrich) was used at a 1:500 dilution. Coverslips were then incubated for 30 min at room temperature with Alexafluor coupled secondary antibodies in blocking buffer and washed an additional three times in blocking buffer. Goat-anti-mouse IgG Alexa-Fluor 647

secondary antibody (A-21235, Thermo Fisher Scientific) was used at a 1:500 dilution. After submerging in ddH2O, cells were mounted onto glass slides using ProLong gold antifade mountant with DAPI (Life Technologies) and visualised using a DeltaVision system (Applied Precision) and deconvolved using SoftWoRx (Applied Precision). Images were processed using ImageJ and OMERO 5.4.10 software (Allan et al., 2012). ImageJ macro quantification of mCh-CK1α spindle localisation was performed as previously described (Fulcher et al., 2019).

QUANTIFICATION AND STATISTICAL ANALYSIS

Statistical analysis was determined using unpaired Student's *t*-test for single comparisons and for multiple treatments analysis of variance was performed followed by the post-hoc tests described in figure legends using Prism® Version 8.0.

Cell Chemical Biology, Volume *27*

Supplemental Information

Inducible Degradation of Target Proteins through a Tractable Affinity-Directed Protein Missile System

Luke M. Simpson, Thomas J. Macartney, Alice Nardin, Luke J. Fulcher, Sascha Röth, Andrea Testa, Chiara Maniaci, Alessio Ciulli, Ian G. Ganley, and Gopal P. Sapkota

Supplemental Information

Inducible degradation of target proteins through a tractable Affinity-directed PROtein Missile (AdPROM) system

Luke M. Simpson, Thomas J. Macartney, Alice Nardin, Luke J. Fulcher, Sascha Röth, Andrea Testa, Chiara Maniaci, Alessio Ciulli, Ian G. Ganley and Gopal P. Sapkota

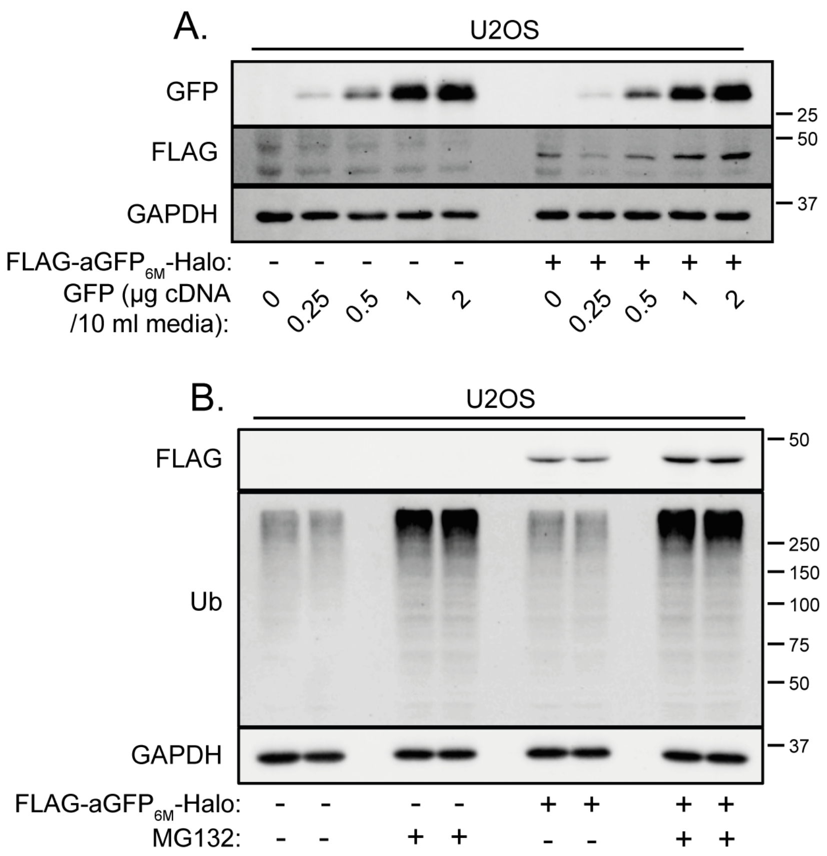

Figure S1, related to Figure 1. FLAG-aGFP$_{6M}$-Halo levels are controlled by GFP protein abundance. (**A**) GFP was transiently expressed with increasing cDNA concentrations (0-2 µg per 10 ml media) in U2OS FLAG-empty and FLAG-aGFP$_{6M}$-Halo expressing cells. (**B**) U2OS FLAG-empty and FLAG-aGFP$_{6M}$-Halo expressing cells were treated with 20 µM MG132 proteasome inhibitor for 6 hr as indicated. For both (**A**) and (**B**), extracts were resolved by SDS-PAGE and transferred on to PVDF membranes, which were subjected to immunoblotting with indicated antibodies.

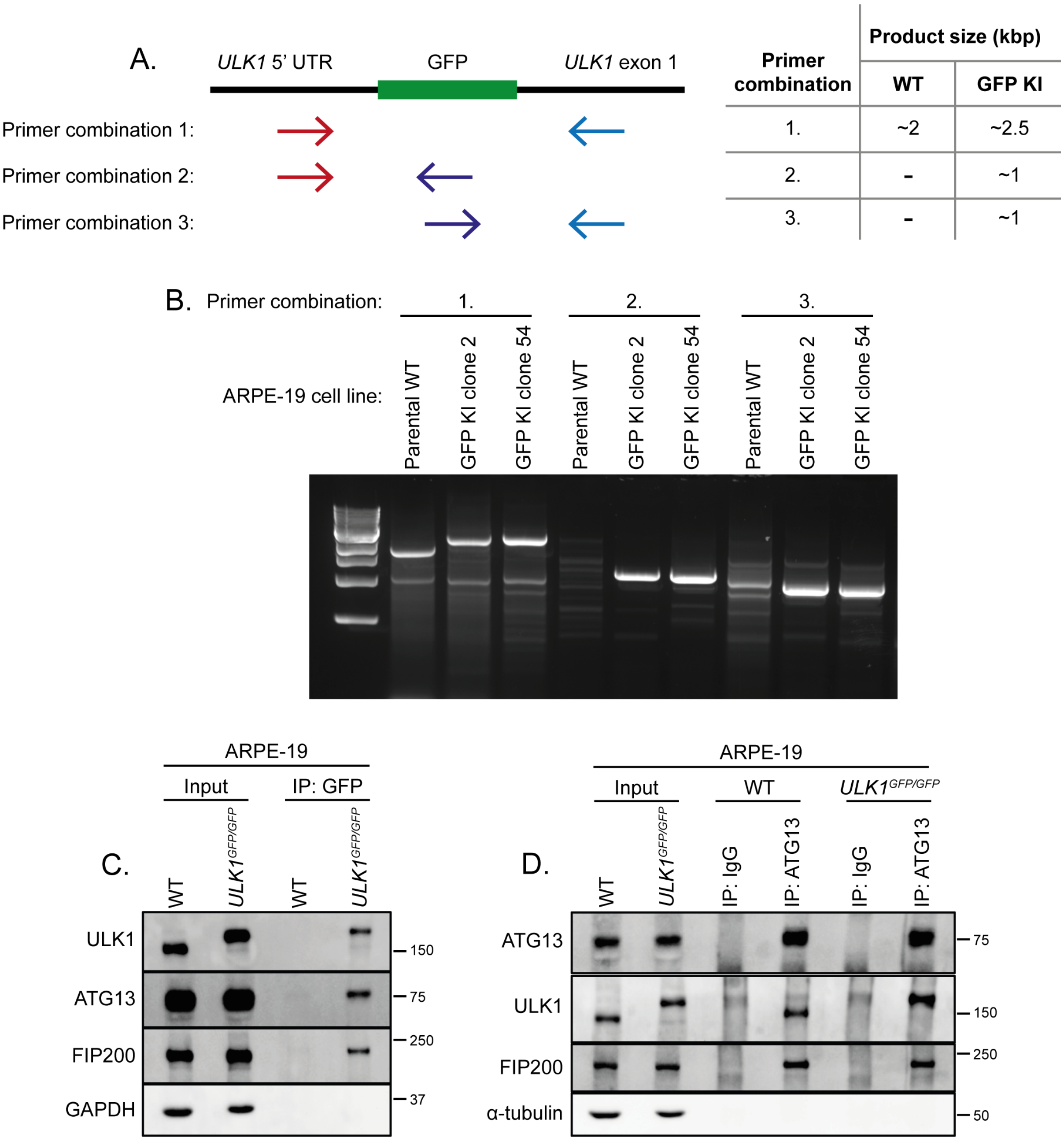

Figure S2, related to Figure 1. Characterisation of ARPE-19 ULK1 GFP knockin (KI) (*ULK1*$^{GFP/GFP}$) cells. (**A**) Primers were designed for analysis of the endogenous *ULK1* locus (primer combination 1) and internal primers to recognise the incorporated GFP-tag (primer combination 2 and 3). (**B**) Agarose gel analysis of ARPE-19 parental WT and ULK1 GFP KI cell line clones 2 and 54. In both clones, an electrophoretic mobility shift is observed in the PCR products of primer combination 1. With both primer combination 2 and 3, no PCR product was observed in parental WT cells, but can be detected for both clone 2 and 54. Clone 54 was used for subsequent experiments. (**C**) ARPE-19 WT and *ULK1*$^{GFP/GFP}$ cells were lysed and subjected to GFP TRAP immunoprecipitation (IP). ULK1 complex components including GFP-ULK1, ATG13 and FIP200 co-precipitated with GFP-ULK1 from *ULK1*$^{GFP/GFP}$ cell extracts. (**D**) ARPE-19 WT and *ULK1*$^{GFP/GFP}$ cells were lysed and subjected to ATG13 or IgG IP as indicated. ULK1 complex components including ATG13, ULK1 and FIP200 co-precipitated with ATG13 from WT and *ULK1*$^{GFP/GFP}$ cell extracts. For both (**C**) and (**D**), extracts and IPs were resolved by SDS-PAGE and transferred on to PVDF membranes, which were subjected to immunoblotting with indicated antibodies.

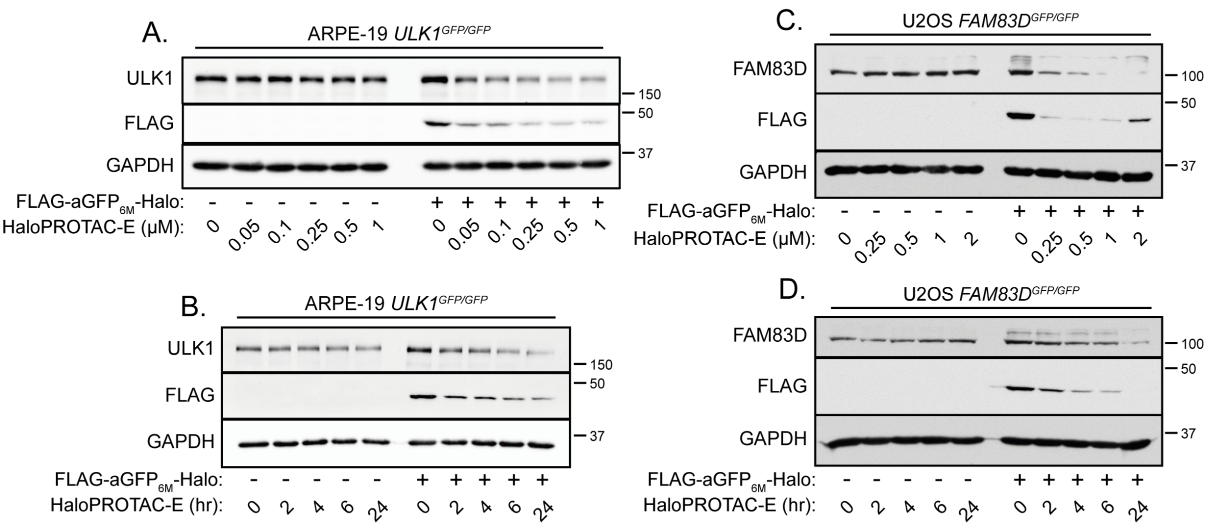

Figure S3, related to Figure 1. GFP-ULK1 and FAM83D-GFP are degraded with HaloPROTAC-E in cells expressing FLAG-aGFP$_{6M}$-Halo. (**A**) ARPE-19 *ULK1$^{GFP/GFP}$* FLAG-empty and FLAG-aGFP$_{6M}$-Halo expressing cells were treated with increasing concentrations of HaloPROTAC-E (0-1 μM) for 24 hr as indicated. (**B**) ARPE-19 *ULK1$^{GFP/GFP}$* FLAG-empty and FLAG-aGFP$_{6M}$-Halo expressing cells were treated with 250 nM HaloPROTAC-E for indicated times (0-24 hr). (**C**) U2OS *FAM83D$^{GFP/GFP}$* FLAG-empty and FLAG-aGFP$_{6M}$-Halo expressing cells were treated with increasing concentrations of HaloPROTAC-E (0-2 μM) for 24 hr. (**D**) U2OS *FAM83D$^{GFP/GFP}$* FLAG-empty and FLAG-aGFP$_{6M}$-Halo expressing cells were treated with 1 μM HaloPROTAC-E for indicated times (0-24 hr). For all experiments, extracts were resolved by SDS-PAGE and transferred on to PVDF membranes, which were subjected to immunoblotting with indicated antibodies.

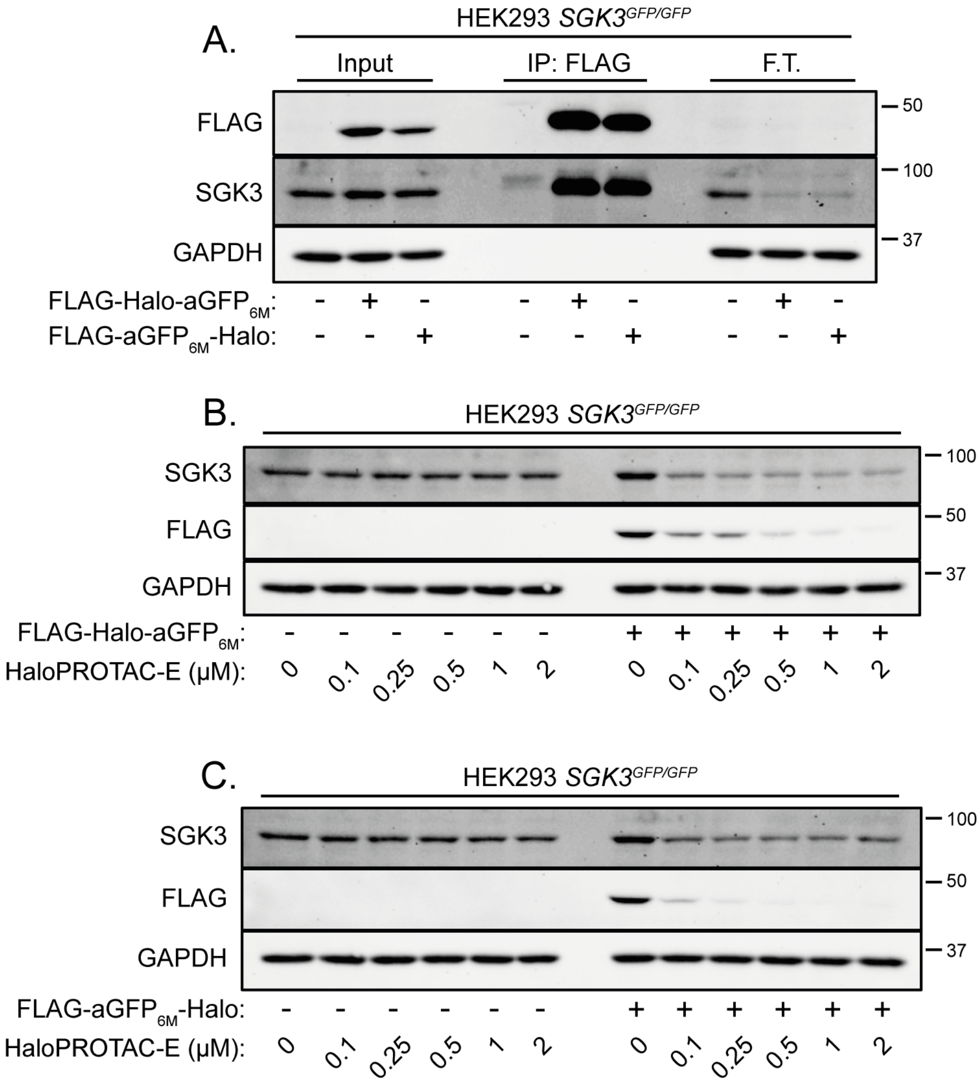

Figure S4, related to Figure 3. SGK3-GFP is degraded with HaloPROTAC-E in FLAG-Halo-aGFP$_{6M}$ and FLAG-aGFP$_{6M}$-Halo expressing cells. (**A**) HEK293 *SGK3*$^{GFP/GFP}$ FLAG-empty, FLAG-Halo-aGFP$_{6M}$ and FLAG-aGFP$_{6M}$-Halo expressing cells were lysed and subjected to immunoprecipitation (IP) with anti-FLAG M2 resin. F.T. = post-IP flow-through extract. HEK293 *SGK3*$^{GFP/GFP}$ FLAG-empty, FLAG-Halo-aGFP$_{6M}$ (**B**) and FLAG-aGFP$_{6M}$-Halo (**C**) expressing cells were treated with increasing concentrations of HaloPROTAC-E (0-2 μM) for 24 hr as indicated. In all cases, extracts and IPs were resolved by SDS-PAGE and transferred on to PVDF membranes, which were subjected to immunoblotting with indicated antibodies.
